# Alteration of the cutaneous microbiome in psoriasis and potential role in Th17 polarization

**DOI:** 10.1186/s40168-018-0533-1

**Published:** 2018-09-05

**Authors:** Hsin-Wen Chang, Di Yan, Rasnik Singh, Jared Liu, Xueyan Lu, Derya Ucmak, Kristina Lee, Ladan Afifi, Douglas Fadrosh, John Leech, Kimberly S. Vasquez, Margaret M. Lowe, Michael D. Rosenblum, Tiffany C. Scharschmidt, Susan V. Lynch, Wilson Liao

**Affiliations:** 1 Department of Dermatology, University of California, San Francisco, CA 94115 USA; 2 Cleveland Clinic Lerner College of Medicine, Cleveland, OH 44106 USA; 3 University of Miami Miller School of Medicine, Miami, FL 33136 USA; 4 Department of Internal Medicine, Yale University, New Haven, CT 06520 USA; 5 Dermatology Department, Peking University Third Hospital, Beijing, China; 6 Department of Dermatology, Dicle University School of Medicine, 21280 Diyarbakır, Turkey; 7 Division of Gastroenterology, University of California, San Francisco, San Francisco, CA 94143 USA

## Abstract

**Background:**

Psoriasis impacts 1–3% of the world’s population and is characterized by hyper-proliferation of keratinocytes and increased inflammation. At the molecular level, psoriasis is commonly driven by a Th17 response, which serves as a major therapeutic target. Microbiome perturbations have been associated with several immune-mediated diseases such as atopic dermatitis, asthma, and multiple sclerosis. Although a few studies have investigated the association between the skin microbiome and psoriasis, conflicting results have been reported plausibly due to the lack of standardized sampling and profiling protocols, or to inherent microbial variability across human subjects and underpowered studies. To better understand the link between the cutaneous microbiota and psoriasis, we conducted an analysis of skin bacterial communities of 28 psoriasis patients and 26 healthy subjects, sampled at six body sites using a standardized protocol and higher sequencing depth compared to previous studies. Mouse studies were employed to examine dermal microbial-immune interactions of bacterial species identified from our study.

**Results:**

Skin microbiome profiling based on sequencing the 16S rRNA V1–V3 variable region revealed significant differences between the psoriasis-associated and healthy skin microbiota. Comparing the overall community structures, psoriasis-associated microbiota displayed higher diversity and more heterogeneity compared to healthy skin bacterial communities. Specific microbial signatures were associated with psoriatic lesional, psoriatic non-lesional, and healthy skin. Specifically, relative enrichment of *Staphylococcus aureus* was strongly associated with both lesional and non-lesional psoriatic skin. In contrast, *Staphylococcus epidermidis* and *Propionibacterium acnes* were underrepresented in psoriatic lesions compared to healthy skin, especially on the arm, gluteal fold, and trunk. Employing a mouse model to further study the impact of cutaneous *Staphylcoccus* species on the skin T cell differentiation, we found that newborn mice colonized with *Staphylococcus aureus* demonstrated strong Th17 polarization, whereas mice colonized with *Staphylococcus epidermidis* or un-colonized controls showed no such response.

**Conclusion:**

Our results suggest that microbial communities on psoriatic skin is substantially different from those on healthy skin. The psoriatic skin microbiome has increased diversity and reduced stability compared to the healthy skin microbiome. The loss of community stability and decrease in immunoregulatory bacteria such as *Staphylococcus epidermidis* and *Propionibacterium acnes* may lead to higher colonization with pathogens such as *Staphylococcus aureus*, which could exacerbate cutaneous inflammation along the Th17 axis.

**Electronic supplementary material:**

The online version of this article (10.1186/s40168-018-0533-1) contains supplementary material, which is available to authorized users.

## Background

Psoriasis is an immune-mediated inflammatory skin disease that impacts 1–3% of the world’s population. The pathogenesis of psoriasis is multifactorial with notable contributions from patient genetics and environmental factors such as lifestyle, diet, and health history [1, 2]. Psoriasis can be mediated by an overactive Th17 response leading to skin inflammation and hyper-proliferation of keratinocytes [3]. In the clinic, blocking components of the Th17 pathway effectively dampens the aberrant immune response in psoriasis patients and controls symptoms, but these treatments do are not curative and disease management effectiveness varies across patients. This highlights the need to further understand the pathogenesis of psoriasis and the factors associated with disease initiation and progression.

The skin is the human body’s largest organ which serves not only as a physical protective barrier against environmental insults, but also as a dynamic interface for host dermal-microbial interactions. The microbial community that inhabits the human skin is highly complex and consists of highly diverse microorganisms including bacteria, fungi, viruses, and archaea [4]. Bacteria have been shown to be essential for skin health by restricting pathogen colonization and fine-tuning resident T cell function [5, 6]. As a result, perturbations to the skin microbial community have the potential to contribute to altered skin immune function. Indeed, dysbiosis of the skin microbiome has been linked to several inflammatory and autoimmune diseases including atopic dermatitis and vitiligo [7, 8], suggesting the importance of the cutaneous microbiome in the health of the skin.

Interestingly, throat and nasal *Streptococcal* infection have been shown to trigger initiation and exacerbation of psoriasis [9, 10], suggesting a microbial contribution to disease. Moreover, keratinocytes, the most prominent cell type in the epidermis, can trigger innate and adaptive immune responses in psoriasis through interactions with skin bacteria [11]. To date, several studies have sought to characterize the psoriasis-associated skin microbiome and identify bacterial species that might contribute to the pathogenesis of psoriasis [12–16]. However, these studies revealed a lack of consensus on psoriasis-associated microbial signatures plausibly due to the inherent heterogeneity of microbial species that promote immune dysfunction in psoriatic patients and or to different study designs. For example, collecting samples using skin swabs [12, 14] or skin biopsies [13] introduces significant variability since these methods sample different cutaneous anatomical compartments with likely different associated bacteria [17]. Moreover, these studies used different 16S rRNA primers amplifying different variable regions of the 16S rRNA gene, which may contribute to variance across studies, making cross study comparisons difficult. Therefore, application of a standardized protocol to allow for a better understanding in the relationship between microbiome and disease is critical [17, 18].

In this study, we surveyed the skin microbiome from 28 psoriasis patients and 26 healthy subjects using the standardized protocol recommended by the NIH Human Microbiome Project [19–21]. In contrast to some previous studies targeting the V4 region of the 16S rRNA gene [13], we profiled the skin microbial community using primers targeting the V1–V3 region, which results in more accurate bacterial identities of the skin microbiome at the genus and species levels compared to the traditional V4 approach [20, 22]. We also used higher sequencing depth to ensure high-quality data. Our data revealed significant alterations in the psoriasis skin microbiome and identified *Staphylococcus aureus* as a potential contributor to psoriasis pathogenesis.

## Results

### Cohort of patients and skin sampling

The cohort in this study consisted of 28 patients with plaque psoriasis and 26 healthy individuals. To avoid any confounding demographic effects, gender and age composition were matched between the two groups (Table 1). All psoriasis patients were clinically diagnosed with psoriasis at the UCSF Psoriasis and Skin Treatment Center. The psoriasis patients in this study had a mean Psoriasis Area and Severity Index (PASI) of 11.1 representing moderate-to-severe disease. To avoid the variabilities introduced by treatments, we excluded subjects with recent antibiotic treatment and/or other biologic and systemic therapy. In addition, all subjects required to undergo a 2-week wash-out period for topical therapy. Different anatomic sites in the human skin can be categorized into three major groups: dry, moist, and sebaceous. The biogeographical differences across different skin sites provide different environments that support distinct microbial communities [23–26]. In order to gain a comprehensive view of the psoriasis-associated skin microbiome, we sampled the microbiome across six different skin sites: scalp, trunk, arm, leg, axilla, and gluteal fold, which covers all three skin groups (Table 2). Three different “disease states” were sampled for each skin site: healthy skin from healthy subjects (Healthy), unaffected or non-lesional skin from psoriasis patients (PSO_N), and lesional skin from psoriasis patients (PSO_L). We sampled all six sites for both healthy (Healthy) and unaffected skin (PSO_N). Only sites with psoriasis lesions present were sampled for psoriatic lesional samples (PSO_L). The psoriasis subjects in our cohort most frequently had psoriatic plaques on the arms, legs, and scalp, whereas there was lowest frequency in the axilla (armpit). Intermediate frequency of plaques was found on the trunk and gluteal fold (Table 2). The sampling of these six skin sites from psoriatic lesional skin, psoriatic non-lesional skin, and healthy control skin allowed for an examination of how the psoriatic microbiome differs at different sites as well as how it changes with disease progression (lesional vs non-lesional).

**Table 1:** Demographic information of study cohort

	Healthy subjects	Psoriasis subjects	*p* value
Sample size	26	28	NA
Mean age (years)	42.3 ± 14.1	43.6 ± 15.1	0.75
Gender (%Female)	46%	61%	0.4132
Mean PASI	NA	11.1 ± 8.9	NA
Median PASI	NA	7.75	NA

*PASI* Psoriasis Area and Severity Index

**Table 2:** Sample composition

Skin site	Arm	Axilla	Scalp	Trunk	Gluteal fold	Leg	Sum
Skin type	Dry	Moist	Sebaceous	Dry	Moist	Dry	–
Healthy	26	19	25	26	25	26	147
PSO_L	22	8	23	17	15	27	112
PSO_N	27	24	25	27	27	28	158

### Alteration in psoriatic skin microbiome diversity is site specific and exhibits an increasing trend in alpha diversity and greater heterogeneity compared with healthy skin

**Fig. 1:**
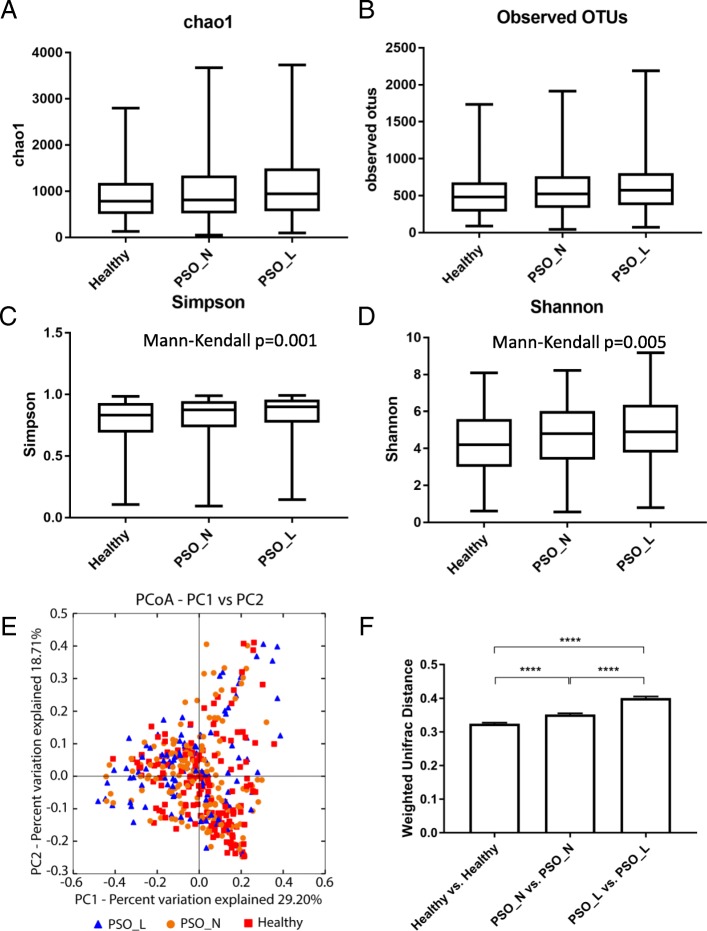
Bacterial community diversity in healthy and psoriasis skin. Alpha diversity measured according to **a** chao1 index, **b** observed OTUs, **c** Simpson’s diversity index, and **d** Shannon index of healthy skin samples, psoriasis non-lesional samples, and psoriasis lesional skin samples. Significant trends of alpha diversity are identified by a Mann-Kendall trend test with *p*-value shown. **e** Principal coordinate analysis (PCoA) of the microbial community structures based on weighted UniFrac distance matrix for the first two principal axes. Each point on the PCoA plot represents a skin microbiome sample (red square = healthy, blue triangle = psoriasis lesional, and orange circle = psoriasis unaffected). The first principal coordinate explains 29.6% of variation, and the second principal coordinate explains 18.70% of the variation. **f** The average weighted UniFrac distances among samples within each disease state are shown in the box plot. The samples in the psoriatic lesional group are more heterogeneous than samples from healthy or psoriasis unaffected groups (one-way ANOVA with Tukey correction, ****p value < 0.0001)

The diversity of the microbial community in a given human body site reflects the structure and composition of the community. Alterations in human microbiome diversity has been linked to disease states. For example, reduced bacterial alpha diversity in the gut microbiome has been linked to obesity and inflammatory bowel disease (IBD) [27–29] while increased diversity in the vaginal microbiome is associated with bacterial vaginosis [30, 31]. To understand if diversity of the skin microbial community is altered in psoriasis patients, we first examined alpha diversity of psoriatic lesional skin, psoriatic unaffected skin and healthy control skin using four different metrics to measure community richness (chao1 and observed OTUs), evenness (Simpson diversity index) and overall diversity (Shannon index) (Fig. 1a–d, and Table 3a). Overall, we observed increasing diversity in all four measures going from healthy skin to non-lesional skin to lesional skin, with a statistically significant trend for the Simpson (Fig. 1c, *p*-value = 0.005) and Shannon indices (Fig. 1d, *p*-value = 0.005). This unidirectional trend in microbiome diversity suggests that the skin microbiome community diversifies as psoriatic disease progresses. To evaluate alpha diversity at different skin sites, we further examined the four metrics at each skin site. Interestingly, we found significantly increased community richness (chao1) in scalp psoriatic lesions compared to healthy scalp and increased community evenness (Simpson and Shannon indices) in arm psoriatic lesional and non-lesional skin compared to arm healthy control skin, with a significant trend test in the arm for the Simpson and Shannon indices (Table 4). When we grouped samples by skin type (Table 5), we observed higher alpha diversity in all four indices at dry psoriatic skin sites (arm, leg, trunk combined) relative to healthy skin, but no difference in alpha diversity for moist sites (axilla, gluteal fold combined). Overall, these results indicate that increased alpha diversity in psoriasis is mostly observed at dry skin sites, with a trend at the sebaceous (scalp) site, and no increase at moist sites. Our data demonstrates that the association between skin microbiome and psoriasis is complex and sometimes site and/or skin type specific. This highlights the need for comprehensive sampling at various skin sites and skin types to study the skin microbiome in association with cutaneous disease.

**Table 3:** Summary of alpha diversity according to disease status

Alpha diversity metrics	Healthy.mean	Healthy.std	PSON.mean	PSON.std	PSOL.mean	PSOL.std	*p* value (trend)
Chao1	891.86	534.25	1012.74	654.40	1090.78	650.49	0.18
Observed OTU	521.77	314.82	572.43	324.16	614.92	346.02	0.15
Shannon	4.33	1.72	4.68	1.70	4.94	1.71	*0.005*
Simpson	0.77	0.20	0.81	0.19	0.84	0.17	*0.00097*

**Table 4:** Summary of alpha diversity within each skin site

Site	Alpha diversity metrics	Healthy.mean	Healthy.std	PSON.mean	PSON.std	PSOL.mean	PSOL.std	*p* value (trend)
Scalp	Chao1	626.17	378.94	794.56	561.43	939.86*	617.36	0.06
Scalp	Observed OTU	335.72	217.12	407.84	287.03	478.3	297.17	0.07
Scalp	Shannon	3.18	1.5	3.38	1.68	3.77	1.59	0.26
Scalp	Simpson	0.68	0.21	0.68	0.21	0.74	0.17	0.47
Arm	Chao1	1024.07	438.77	1381.73	742.52	1255.45	680.35	0.19
Arm	Observed OTU	594.58	220.89	772.44	328.77	755.73	400.6	0.11
Arm	Shannon	4.43	1.42	5.44*	1.44	5.66**	1.54	*0.007*
Arm	Simpson	0.76	0.17	0.86*	0.14	0.89*	0.11	*0.002*
Leg	Chao1	1169.15	558.4	1351.63	780.77	1356.34	735.23	0.4
Leg	Observed OTU	687.62	300.84	764.07	359.84	763.3	364.11	0.37
Leg	Shannon	5.37	1.29	5.68	1.43	5.49	1.7	0.59
Leg	Simpson	0.89	0.08	0.89	0.11	0.86	0.17	0.54
Trunk	Chao1	880.93	506.25	950.95	537.29	1038.19	469.78	0.28
Trunk	Observed OTU	518.85	312.88	540.48	277.26	609.47	238.09	0.17
Trunk	Shannon	4.07	2.07	4.32	1.87	5.19	1.42	0.09
Trunk	Simpson	0.69	0.27	0.75	0.25	0.86	0.14	0.07
Axilla	Chao1	610.81	489.85	676.19	459.89	544.68	475.22	0.89
Axilla	Observed OTU	357.21	283.61	390.33	225.85	285.88	175.58	0.82
Axilla	Shannon	3.78	1.49	3.99	1.19	3.62	1.15	0.19
Axilla	Simpson	0.76	0.16	0.78	0.16	0.78	0.24	0.22
Gluteal fold	Chao1	956.62	572.91	855.25	354.03	953.51	412.17	0.78
Gluteal fold	Observed OTU	587.72	366.3	519.89	193.96	532.47	204.86	0.85
Gluteal fold	Shannon	5.01	1.38	5.04	1.23	5.12	1.36	0.91
Gluteal fold	Simpson	0.85	0.12	0.87	0.1	0.88	0.1	0.22

*p value < 0.05 compared to healthy, **p value < 0.01 compared to healthy

**Table 5:** Summary of alpha diversity according to skin type

Skin type	Metrics	Healthy.mean	Healthy.std	PSON.mean	PSON.std	PSOL.mean	PSOL.std	*p* value (trend)
Dry	Chao1	1024.72	517.09	1229.61	723.2	1240.76	669.61	*0.05*
Dry	Observed OTU	600.35	289.53	693.21	341.44	721.15	355.91	*0.02*
Dry	Shannon	4.62	1.72	5.15	1.7	5.47	1.59	*0.004*
Dry	Simpson	0.78	0.21	0.83	0.19	0.87	0.15	*0.001*
Moist	Chao1	807.29	565.2	770.99	416.98	811.31	476.72	0.89
Moist	Observed OTU	488.18	352.14	458.92	219.32	446.7	227.79	0.86
Moist	Shannon	4.48	1.55	4.55	1.32	4.6	1.48	0.5
Moist	Simpson	0.81	0.15	0.83	0.14	0.85	0.17	0.25

Dry = arm, leg, trunk; moist = axilla, gluteal fold

We further explored the relationship among bacterial communities isolated from psoriatic and healthy skin by calculating beta diversity using weighted Unifrac distance [32]. There was no distinct difference between bacterial communities isolated from the healthy skin and psoriatic skin as there was not a distinctive separation between bacterial communities isolated according to skin status (Fig. 1c) and the first PC is not significantly different in both psoriasis disease states and healthy skin (PSO_L vs Healthy: *p* value = 0.109, PSO_N vs. Healthy: *p* value = 0.128). Although we did not observe distinct clusters associated with disease states, the bacterial communities isolated from psoriatic skin were more dispersed in the principal coordinate analysis than those from healthy skin (Fig. 1e). Indeed, we assess the community dispersion of each disease status by calculating the mean weighted Unifrac distance between bacterial microbiota found in either healthy, psoriatic non-lesional or psoriatic lesional skin and noted that psoriatic non-lesional skin or psoriatic lesional skin exhibited significantly higher mean distances compared with healthy skin (Fig. 1f), indicating greater heterogeneity in the composition of skin microbiota of psoriatic patients irrespective of lesions. We observed a similar trend of increasing heterogeneity by disease state in skin bacterial communities isolated from the arm, trunk, and leg (Additional file 1: Figure S1A, S1B, S1C) as well as in the dry skin group (Additional file 1: Figure S2A). In the moist skin group, bacterial communities of psoriasis lesional skin also exhibited higher heterogeneity compared to healthy and non-lesional skin (Additional file 1: Figure S2B). The heterogeneity differences in moist skin group were largely driven by samples from the gluteal fold (Additional file 1: Figure S1E), as there was little difference in heterogeneity for the axilla (Additional file 1: Figure S1D). Interestingly, the scalp skin microbiome displayed no differences in heterogeneity among different disease states. These results indicate increasing beta heterogeneity for all dry skin sites in psoriasis and for the gluteal fold in psoriasis. Taken with the previous results for alpha diversity, there appears to be an overall loss of stability in the skin microbial community as psoriatic disease progresses, particularly for dry skin sites.

### Psoriasis skin microbiota is enriched for *Staphylococcus aureus* and *Staphylococcus pettenkoferi*

**Fig. 2:**
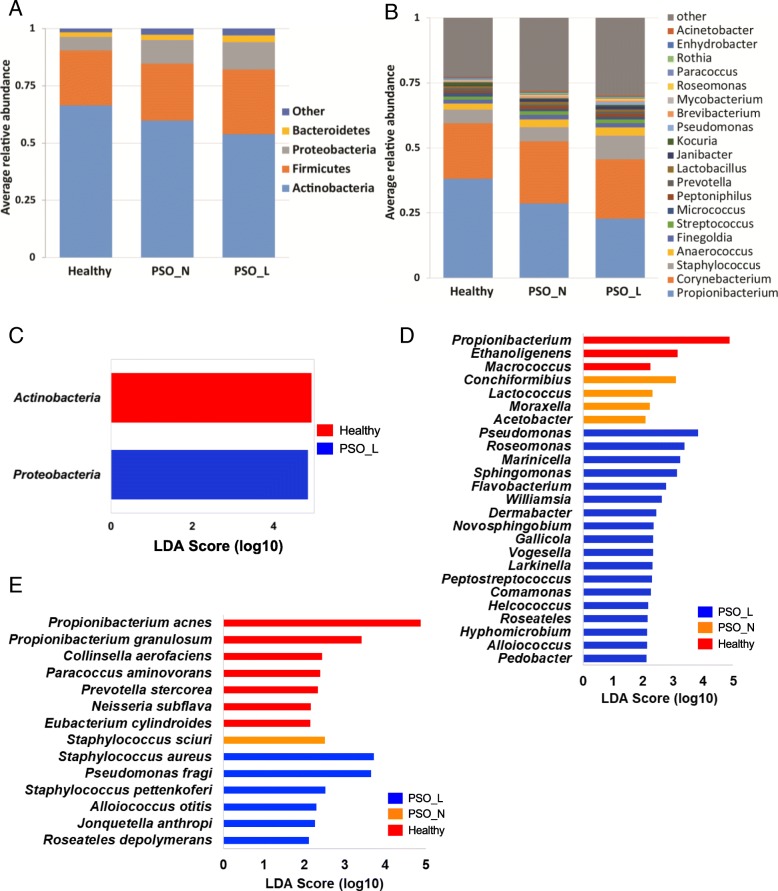
Taxonomical compositions and microbial signatures associated with each disease state. **a** Phylum and **b** genus level compositions of skin microbiome in healthy skin (Healthy), psoriasis unaffected skin (PSO_N), and psoriatic lesional skin (PSO_L). Only the predominant taxa are shown. Other represents lower abundant taxa that are not plotted. Bacterial taxa that are enriched in samples from healthy skin (red), psoriatic lesional skin (blue: PSO_L), and psoriatic unaffected skin (orange: PSO_N) at **c** phylum, **d** genus, and **e** species level. No phyla were significantly enriched in psoriasis unaffected skin

We next examined bacterial composition of skin microbial communities from psoriatic and healthy skin at various taxonomic levels. The skin microbiome of all disease states (healthy, psoriatic lesional, and psoriatic non-lesional) consisted of four dominant phyla: *Actinobacteria* (53.8–66.5%) *Firmicutes* (23.9–28.3%), *Proteobacteria* (5.8–12.0%), and *Bacteroidetes* (2.1–2.9%) (Fig. 2a), consistent with previous descriptions of skin microbiota composition [4]. At the genus level, skin microbiome is dominated by *Propionibacterium* (22.8–38.1%), *Corynebacterium* (21.4–23.9%), *Staphylococcus* (5.3–9.2%) in all disease states (Fig. 2b)*.* Although the dominant taxa are similar in different disease states, we observed a gradual shift of taxonomic composition from healthy skin to psoriatic non-lesional skin to psoriatic lesional skin at both phylum and genus levels, suggesting that these microbial community shifts may precede the appearance of lesions in patients and have potential roles in disease progression. To further associate the taxonomic shift to different disease states, we identified bacterial taxa that discriminate each disease group using Lefse [33]. At the phylum level, *Actinobacteria* and *Proteobacteria* served as strong discriminants for the skin microbiome from healthy and psoriatic lesions respectively (Fig. 2c). Lefse identified three genera, *Propionibacterium*, *Ethanoligenens*, and *Macrococcus*, as additional discriminative signatures for healthy skin (Fig. 2d). Lefse also identified 18 microbial genera that are discriminatively associated with psoriatic lesional skin including the genus *Pseudomonas*, which includes many opportunistic pathogens (Fig. 2d, Table 6). Four genera, *Conchiformibius*, *Lactococcus*, *Moraxella*, and *Acetobacter*, were associated with psoriatic unaffected skin (Fig. 2d). The combination of these genera can serve as potential markers for distinguishing skin from different disease states.

**Table 6:** Microbial genera associated with different skin status

Feature	Log(highest_class_avg)	Class enriched	LDA effect size	*p* value
g__Propionibacterium	5.58	Healthy	4.88	1.54E−04
g__Ethanoligenens	0.61	Healthy	3.16	2.47E−02
g__Macrococcus	2.60	Healthy	2.25	2.37E−02
g__Pseudomonas	3.99	PSO_L	3.83	2.04E−04
g__Roseomonas	3.77	PSO_L	3.38	3.35E−02
g__Marinicella	1.11	PSO_L	3.24	1.65E−02
g__Sphingomonas	3.54	PSO_L	3.14	5.05E−03
g__Flavobacterium	3.16	PSO_L	2.78	2.87E−05
f__Flavobacteriaceae_Other	1.03	PSO_L	2.67	4.15E−03
g__Williamsia	3.05	PSO_L	2.63	4.97E−02
f__Micrococcaceae_Other	3.23	PSO_L	2.60	8.07E−03
g__Dermabacter	3.25	PSO_L	2.45	3.27E−03
g__Novosphingobium	3.00	PSO_L	2.36	8.24E−03
g__gallicola	2.68	PSO_L	2.34	3.96E−02
g__Vogesella	2.50	PSO_L	2.34	2.36E−02
g__Larkinella	1.16	PSO_L	2.32	6.12E−03
g__Peptostreptococcus	2.86	PSO_L	2.29	4.72E−02
g__Comamonas	2.67	PSO_L	2.27	2.11E−02
f__Rhodocyclaceae_Other	2.78	PSO_L	2.22	4.96E−02
g__Helcococcus	2.61	PSO_L	2.17	9.83E−04
g__Roseateles	0.83	PSO_L	2.16	2.08E−02
g__Hyphomicrobium	2.56	PSO_L	2.13	1.86E−02
g__Alloiococcus	2.54	PSO_L	2.13	1.43E−06
g__Pedobacter	2.60	PSO_L	2.13	1.90E−02
g__Conchiformibius	3.28	PSO_N	3.10	6.96E−04
f__Bradyrhizobiaceae_Other	2.99	PSO_N	2.66	2.30E−02
g__Lactococcus	2.98	PSO_N	2.32	1.58E−02
g__Moraxella	2.55	PSO_N	2.22	4.54E−03
g__Acetobacter	2.25	PSO_N	2.08	2.59E−03

**Fig. 3:**
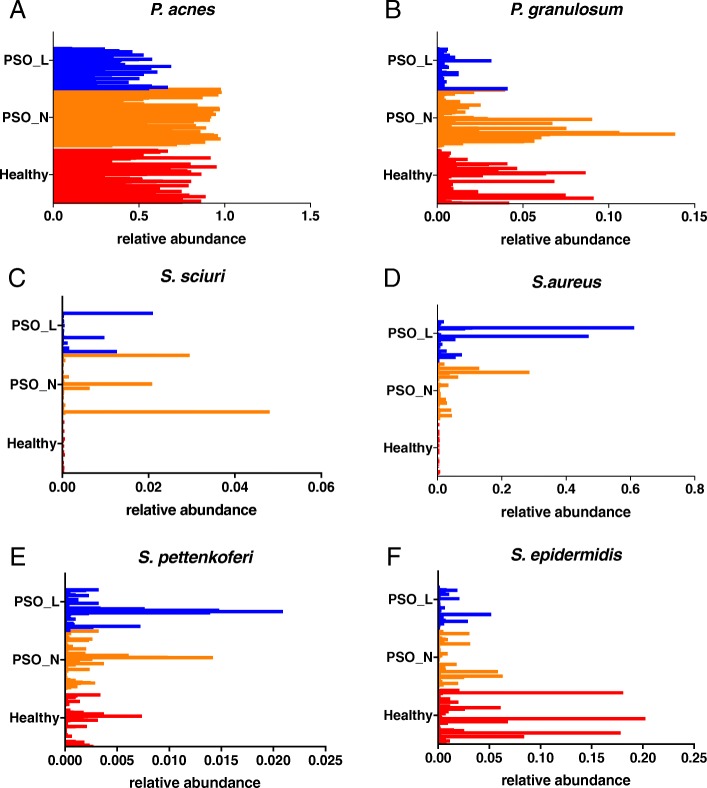
Relative abundance of bacterial species in each disease state. Histograms represent the relative abundances of specific bacterial species in samples from healthy skin (red bars: Healthy), psoriatic lesions (blue bars: PSO_L), and psoriatic unaffected skin (orange bars: PSO_N). Samples from healthy skin and psoriatic unaffected skin are more abundant in **a** *Propionibacterium acnes* (*p* value = 0.0002; LDA effect size = 4.87) and **b** *Propionibacterium granulosum* (*p* value = 0.014; LDA effect size = 3.41). Samples from psoriatic skin (both unaffected and lesional) are more abundant in **c** *Staphylococcus sciuri* (*p* value = 0.032; LDA effect size = 2.51), **d** *Staphylococcus aureus* (*p* value = 0.007; LDA effect size = 3.72), and **e** *Staphylococcus pettenkoferi* (*p* value = 0.012; LDA effect size = 2.52)*.* On the contrary, **f** *Staphylococcus epidermidis* shows a trend of increased abundance in healthy skin but the difference did not reach the statistical significance

Our 16S rRNA sequencing also provided species-level resolution for some but not all of the sequencing reads. Lefse analysis identified several species-level bacterial signatures specific for different disease states (Fig. 2e). Consistent with what we observed in the genus level, the healthy skin microbiome was more enriched in both *Propionibacterium acnes* (*P*. *acnes*) and *Propionibacterium granulosum* (*P*. *granulosum*) compared to the psoriasis-associated skin microbiome (Fig. 3a, b). *Staphylococcus sciuri* was enriched in psoriatic non-lesional skin (Fig. 3c). Interestingly, two *Staphylococcus* species, *S*. *aureus* and *S*. *pettenkoferi* were significantly enriched in the psoriatic lesions while the genera *Staphylococcus* as a whole was not significantly enriched with any skin condition in our analysis (Fig. 2d, e).

We further explored the relative abundance of the *Staphylococcus* species across all samples with different disease states (Table 7). Strikingly, *Staphylococcus aureus* was more abundant in both lesional and non-lesional psoriatic skin compared to healthy skin (Fig. 3d). Although a low level of *S*. *aureus* was detected in 102 out of 147 healthy control samples and was detected in at least one skin swab of every healthy control subject, increased *S*. *aureus* abundance was exclusively observed in psoriasis samples (Fig. 3d). A similar trend was observed for *Staphylococcus pettenkoferi*, although to a lesser degree (Fig. 3e). In contrast, *Staphylococcus epidermidis* was more abundant in healthy skin compared to psoriatic skin (Fig. 3f) which is consistent with the previously reported competitive relationship between the *Staphylococcus epidermidis and Staphylococcus aureus* [34]*.* The dynamic inter-microbe relationship between different *Staphylococcus* species might contribute to the distinct microbial communities associated with healthy and psoriatic skin.

**Table 7:** Microbial species associated with different skin status

Feature	Log(highestClassAvg)	Class	LDA effect size	*p* value
f__Propionibacteriaceae_g__Propionibacterium_s__acnes	5.57	Healthy	4.87	1.92E−04
f__Propionibacteriaceae_g__Propionibacterium_s__granulosum	3.96	Healthy	3.41	1.36E−02
f__Coriobacteriaceae_g__Atopobium_other	3.32	Healthy	2.93	6.00E−03
f__Ruminococcaceae_g__Ethanoligenens_s__	0.61	Healthy	2.66	2.47E−02
f__Gracilibacteraceae_g___s__	0.67	Healthy	2.48	1.76E−02
f__Coriobacteriaceae_g__Collinsella_s__aerofaciens	2.89	Healthy	2.44	1.09E−02
f__Sphingomonadaceae_g__Novosphingobium_s__	2.99	Healthy	2.43	1.07E−02
f__Rhodobacteraceae_g__Paracoccus_s__aminovorans	2.80	Healthy	2.39	1.00E−02
f__Prevotellaceae_g__Prevotella_s__stercorea	2.60	Healthy	2.33	1.71E−03
f__Desulfovibrionaceae_g__Desulfovibrio_s__	1.16	Healthy	2.24	2.47E−02
f__Rickettsiaceae_g__Rickettsia_s__	1.40	Healthy	2.22	2.83E−03
f__Victivallaceae_g___s__	1.04	Healthy	2.20	6.97E−03
f__Rivulariaceae_other_other	1.06	Healthy	2.19	2.47E−02
f__Erysipelotrichaceae_g__cc_115_s__	0.94	Healthy	2.16	1.71E−02
f__Neisseriaceae_g__Neisseria_s__subflava	2.68	Healthy	2.16	1.83E−02
f__Erysipelotrichaceae_g___Eubacterium__s__cylindroides	2.34	Healthy	2.15	1.71E−02
f__Succinivibrionaceae_g__Succinivibrio_s__	1.88	Healthy	2.06	5.69E−04
f__Lachnospiraceae_g__Coprococcus_other	1.55	Healthy	2.01	6.91E−08
f__Staphylococcaceae_g__Staphylococcus_s__aureus	4.16	PSO_L	3.72	7.47E−03
f__Pseudomonadaceae_g__Pseudomonas_s_fragi	3.93	PSO_L	3.64	3.35E−04
f__Methylobacteriaceae_g___s__	3.81	PSO_L	3.38	1.68E−03
f__oxalobacteraceae_g___s__	3.69	PSO_L	3.27	4.40E−03
f__flavobacteriaceae_other_other	1.03	PSO_L	2.94	4.15E−03
f__Sphingomonadaceae_g__Sphingomonas_s__	3.41	PSO_L	2.94	2.08E−02
o__Thiohalorhabdales_f___g___s__	1.40	PSO_L	2.89	4.15E−03
f__Ellin517_g___s__	0.48	PSO_L	2.85	2.28E−02
f___Marinicellaceae__g__Marinicella_s__	1.11	PSO_L	2.85	1.65E−02
f__Propionibacteriaceae_g__Tessaracoccus_s__	0.78	PSO_L	2.79	2.76E−02
f___g___s__	1.08	PSO_L	2.77	2.28E−02
f__flavobacteriaceae_g__flavobacterium_s__	3.12	PSO_L	2.69	5.19E−05
f__Micrococcaceae_other_other	3.23	PSO_L	2.69	9.67E−03
f__Williamsiaceae_g__Williamsia_s__	3.05	PSO_L	2.68	4.36E−02
f__Microbacteriaceae_g___s__	3.27	PSO_L	2.67	2.24E−02
f__Xanthomonadaceae_g__Wohlfahrtiimonas_s__	1.16	PSO_L	2.66	1.65E−02
f__Cytophagaceae_g__Larkinella_s__	1.16	PSO_L	2.60	6.02E−03
f__Pseudomonadaceae_g__Pseudomonas_s__	3.06	PSO_L	2.57	2.33E−03
f__Rhodobacteraceae_g__Anaerospora_other	1.16	PSO_L	2.52	5.25E−03
f__Staphylococcaceae_g__Staphylococcus_s__pettenkoferi	2.99	PSO_L	2.52	1.23E−02
f__Comamonadaceae_g__Limnobacter_s__	1.55	PSO_L	2.51	4.31E−02
f__Kineosporiaceae_g__Kineosporia_s__	1.26	PSO_L	2.49	3.75E−02
f__Legionellaceae_g__Legionella_other	1.60	PSO_L	2.46	1.15E−02
f__Chitinophagaceae_g___s__	2.81	PSO_L	2.44	4.84E−04
f__Acetobacteraceae_g___s__	3.11	PSO_L	2.42	1.75E−02
f__frankiaceae_other_other	1.46	PSO_L	2.42	1.91E−03
f__Ectothiorhodospiraceae_g___s__	1.67	PSO_L	2.36	1.85E−02
f__Dermabacteraceae_g__Dermabacter_s__	3.25	PSO_L	2.34	2.79E−03
f__Coxiellaceae_g___s__	1.60	PSO_L	2.31	2.62E−02
f___Tissierellaceae__g__Gallicola_s__	2.68	PSO_L	2.30	3.94E−02
f__Aerococcaceae_g__Alloiococcus_s_otitis	2.41	PSO_L	2.30	1.04E−05
f__Hyphomicrobiaceae_g__Hyphomicrobium_s__	2.52	PSO_L	2.28	2.71E−02
f__Dethiosulfovibrionaceae_g__Jonquetella_s__anthropi	0.99	PSO_L	2.26	2.08E−02
f__Rhodospirillaceae_g___s__	2.66	PSO_L	2.26	1.26E−02
f__Comamonadaceae_g__Comamonas_s__	2.54	PSO_L	2.24	4.73E−03
f__Piscirickettsiaceae_g___s__	1.78	PSO_L	2.23	4.39E−03
f__Methylobacteriaceae_g__Methylobacterium_s__	2.86	PSO_L	2.21	1.00E−02
f___Tissierellaceae__g__Helcococcus_s__	2.61	PSO_L	2.21	1.05E−03
f__Comamonadaceae_g__Rhodoferax_s__	1.42	PSO_L	2.18	1.39E−02
f__Neisseriaceae_g__Vogesella_s__	2.50	PSO_L	2.18	2.42E−02
f__Leuconostocaceae_g__Weissella_other	1.33	PSO_L	2.17	9.64E−03
f__Alcaligenaceae_g_oligella_s__	2.34	PSO_L	2.12	3.31E−02
f__Erythrobacteraceae_g___s__	2.67	PSO_L	2.12	2.31E−03
f__Beijerinckiaceae_g___s__	2.54	PSO_L	2.12	1.62E−02
f__Comamonadaceae_g__Roseateles_s__depolymerans	0.83	PSO_L	2.12	2.08E−02
f__Bacillaceae_other_other	2.27	PSO_L	2.07	8.81E−04
o__Phycisphaerales_f___g___s__	1.59	PSO_L	2.04	3.64E−02
f__Neisseriaceae_g___s__	4.28	PSO_N	3.78	2.89E−02
f__Neisseriaceae_g__Conchiformibius_s__	3.28	PSO_N	3.08	4.83E−05
f__Moraxellaceae_g__Acinetobacter_other	3.26	PSO_N	2.95	9.18E−03
f__Micrococcaceae_g___s__	3.34	PSO_N	2.81	5.11E−03
f__Bradyrhizobiaceae_other_other	2.99	PSO_N	2.70	2.23E−02
f__Staphylococcaceae_g__Staphylococcus_s__sciuri	2.85	PSO_N	2.51	3.23E−02
f__Syntrophobacteraceae_g___s__	1.26	PSO_N	2.40	2.85E−02
f__Streptococcaceae_g__Lactococcus_s__	2.98	PSO_N	2.38	3.17E−02
f___Chthoniobacteraceae__g___s__	1.26	PSO_N	2.35	2.55E−02
f__Moraxellaceae_g__Moraxella_s__	2.55	PSO_N	2.29	1.29E−03
f__Moraxellaceae_g__Perlucidibaca_s__	2.06	PSO_N	2.25	2.62E−02
f__Actinomycetaceae_g__Trueperella_s__	1.01	PSO_N	2.22	3.63E−02
f__Pseudonocardiaceae_g___s__	1.09	PSO_N	2.08	2.21E−02

Anatomic skin site is one of the major determinants of skin microbiome composition [24, 35]. Therefore, we further used Lefse to identify bacterial species at each skin site associated with healthy, non-lesional psoriatic, and lesional psoriatic skin (Table 8). We found that a reduced abundance of *P*. *acnes* is associated with psoriasis lesional skin at the arm, trunk, and gluteal fold (Additional file 1: Figure S3A), with a similar trend for the scalp and axilla. We did not observe a decrease for *P*. *acnes* in leg psoriasis samples, which is possibly due to the low abundance of *P*. *acnes* in healthy leg skin (Additional file 1: Figure S3A). Together, our data suggest that *P*. *acnes* may play a crucial role to maintain skin health at most skin sites besides the leg. Surprisingly, we did not observe a statistically significant increase in *S*. *aureus* abundance in psoriasis compared to healthy skin at any individual skin site (Table 8), whereas when anatomic sites were combined, *S*. *aureus* was highly associated with psoriasis lesional skin (Figs. 2e and 3d). Therefore, we defined a group of psoriasis samples with *S*. *aureus* abundance above the highest level of *S*. *aureus* colonization in healthy skin (baseline level = 0.0068) as “*S*. *aureus* high samples” (Table 9). We found that *S*. *aureus* high samples were observed exclusively in psoriasis patients and were seen at all skin sites (Additional file 1: Figure S3C), but that the number of *S*. *aureus* high samples at each skin site is modest, between 2 and 8 (Additional file 1: Figure S3D). This indicates that the association of *S*. *aureus* with psoriasis is not driven by any single anatomic site and that the presence of abundant *S*. *aureus* in only a subset of psoriasis patients, at different anatomic locations, results in an underpowered sample size for detection of *S*. *aureus* at any single body site.

**Table 8:** Microbial species associated with different disease state in each skin site

Feature	Log(highest_class_avg)	Class enriched	LDA effect size	*p* value
Arm				
Propionibacterium_acnes	5.67	Healthy	5.02	0.001
Leadbetterella_s__	1.54	PSO_L	2.25	0.024
Comamonas_s__	2.94	PSO_L	2.59	0.041
Acinetobacter_Other	3.44	PSO_L	3.13	0.048
Vogesella_s__	3.20	PSO_L	3.03	0.047
Conchiformibius_s__	4.02	PSO_N	3.61	0.047
Pseudomonas_s__	3.30	PSO_N	2.83	0.035
Peptococcus_s__	2.26	PSO_N	2.00	0.019
Euzebya_s__	2.14	PSO_N	2.03	0.038
Trunk				
Propionibacterium_s__acnes	5.68	Healthy	5.19	0.004
Moraxella_s__	2.51	PSO_L	2.29	0.006
Helcococcus_s__	2.50	PSO_L	2.14	0.006
Conchiformibius_s__	2.53	PSO_N	2.26	0.027
Leg				
Xanthobacter_s__	2.31	Healthy	2.06	0.011
Flavobacterium_s__	2.93	PSO_L	2.38	0.028
Pseudomonas_s__fragi	3.25	PSO_L	2.88	0.036
Pseudomonas_Other	2.38	PSO_L	2.00	0.008
Axilla				
Selenomonas_s__noxia	1.25	Healthy	2.13	0.028
Paracoccus_s__aminovorans	2.61	Healthy	2.37	0.006
Lactobacillus_s__	3.19	Healthy	2.94	0.019
Propionibacterium_Other	1.33	PSO_L	2.09	0.041
Bradyrhizobium_s__	1.55	PSO_N	2.16	0.048
Methylopila_s__	2.33	PSO_N	2.13	0.048
Veillonella_s__dispar	2.71	PSO_N	2.44	0.013
Peptostreptococcus_s__	1.50	PSO_N	2.15	0.048
Rhodococcus_s__	2.28	PSO_N	2.09	0.015
Streptococcus_s__	3.84	PSO_N	3.48	0.021
Gluteal fold				
Propionibacterium_s__acnes	5.38	Healthy	4.78	0.043
Mycobacterium_Other	3.65	Healthy	3.33	0.002
Propionibacterium_s__granulosum	3.59	Healthy	3.07	0.031
Mitsuokella_s__	2.64	Healthy	2.55	0.012
Amaricoccus_s__	2.73	Healthy	2.42	0.043
Mycobacterium_s__vaccae	2.04	Healthy	2.12	0.018
Scalp				
Flavobacterium_s__	2.58	PSO_L	2.17	0.001
Pseudomonas_s__fragi	3.09	PSO_L	2.70	0.011
Pseudomonas_s__	2.73	PSO_N	2.22	0.025
Sphingomonas_s__	3.15	PSO_N	2.78	0.006
Staphylococcus_Other	3.41	PSO_N	2.87	0.040

**Table 9:** Sample information of *S*. *aureus* high samples

#PID	Skin_type	Skin_site	Disease state	*S*. *aureus*_abundance	Baseline	Fold change
7319	Sebaceous	Scalp	PSO_L	0.6119	0.0068	90.0
7314	Dry	Leg	PSO_L	0.4699	0.0068	69.1
7319	Sebaceous	Scalp	PSO_N	0.2857	0.0068	42.0
7319	Dry	Trunk	PSO_N	0.1300	0.0068	19.1
7306	Sebaceous	Scalp	PSO_L	0.1085	0.0068	16.0
7331	Sebaceous	Scalp	PSO_L	0.0863	0.0068	12.7
7331	Dry	Arm	PSO_L	0.0760	0.0068	11.2
7313	Sebaceous	Scalp	PSO_N	0.0647	0.0068	9.5
7319	Dry	Arm	PSO_L	0.0569	0.0068	8.4
7331	Dry	Leg	PSO_L	0.0559	0.0068	8.2
7319	Dry	Arm	PSO_N	0.0454	0.0068	6.7
7331	Dry	Arm	PSO_N	0.0424	0.0068	6.2
7331	Sebaceous	Scalp	PSO_N	0.0378	0.0068	5.6
7331	Dry	Leg	PSO_N	0.0344	0.0068	5.1
7331	Moist	Axilla	PSO_N	0.0295	0.0068	4.3
7331	Moist	Axilla	PSO_L	0.0288	0.0068	4.2
7319	Moist	Gluteal_fold	PSO_N	0.0260	0.0068	3.8
7331	Dry	Trunk	PSO_N	0.0219	0.0068	3.2
7331	Dry	Trunk	PSO_L	0.0201	0.0068	3.0
7319	Dry	Leg	PSO_L	0.0157	0.0068	2.3
7306	Dry	Arm	PSO_L	0.0120	0.0068	1.8
7306	Dry	Trunk	PSO_L	0.0118	0.0068	1.7
7302	Sebaceous	Scalp	PSO_L	0.0116	0.0068	1.7
7327	Dry	Leg	PSO_L	0.0106	0.0068	1.6
7319	Dry	Leg	PSO_N	0.0104	0.0068	1.5
7306	Moist	Gluteal_fold	PSO_N	0.0101	0.0068	1.5
7331	Moist	Gluteal_fold	PSO_N	0.0100	0.0068	1.5
7314	Moist	Gluteal_fold	PSO_L	0.0089	0.0068	1.3
7305	Dry	Arm	PSO_N	0.0085	0.0068	1.3
7306	Dry	Leg	PSO_L	0.0080	0.0068	1.2
7306	Dry	Leg	PSO_N	0.0078	0.0068	1.1
7309	Dry	Arm	PSO_N	0.0070	0.0068	1.0

### Correlations between different bacterial species

**Fig. 4:**
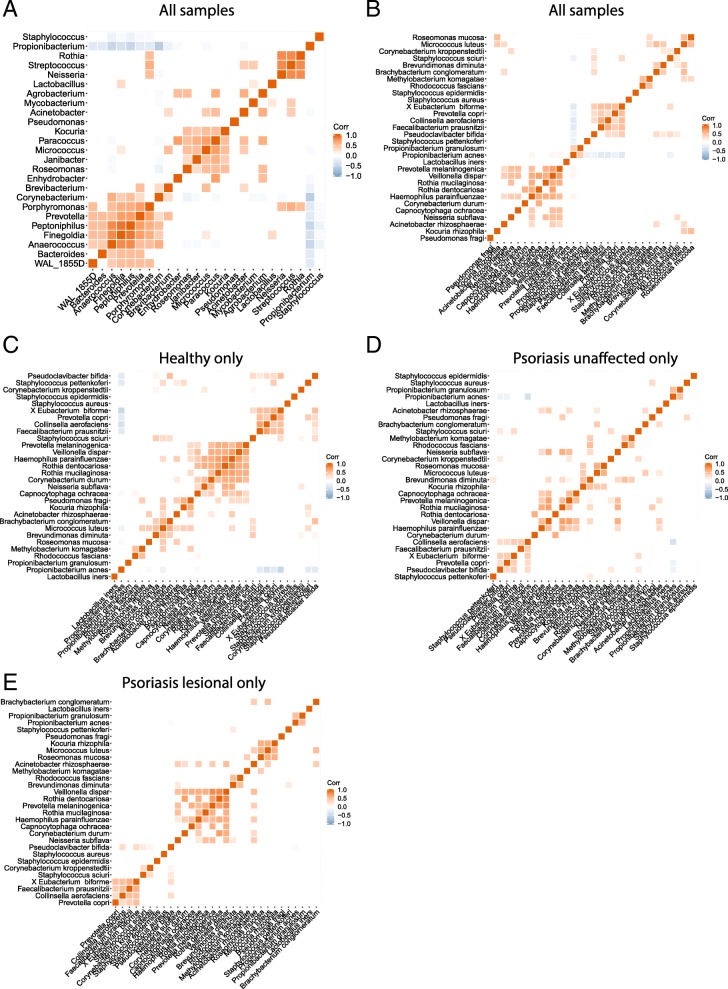
Correlations between the most abundant bacterial genera and species. Correlation plots show the Spearman correlations among **a** the top 25 most abundant genera or **b** the top 30 most abundant species in all samples. Correlations among the top 30 most abundant bacterial species associated with **c** Healthy skin samples, **d** psoriasis unaffected samples, and **e** psoriasis lesional samples. Only the correlations with statistical significance are shown. Color and intensity indicate directions and strength of the correlation

Like any ecosystem, the composition of skin microbiome is modulated by both environmental factors (i.e., nutrient availability and host immune response) and interactions between different bacterial species. Inter-microbial interactions can be a major driver of microbial community composition, and understanding this interaction can yield important insights regarding the establishment and maintenance of psoriasis-associated microbial communities. We further investigated this microbe-microbe interaction by correlating microbial abundances with each other. At the genus level, we identified three clusters of bacterial communities, each constituting a group of bacteria significantly correlated in abundance (Fig. 4a). Cluster A was the largest cluster and consisted of *Corynebacterium*, *Porphyromonas*, *Prevotella*, *Peptoniphilus*, *Finegoldia*, and *Anaercoccus*. Cluster B was composed of *Kocuria*, *Paracoccus*, *Micrococcus*, and *Janibacter*. Lastly, Cluster C consisted of strongly correlated *Streptococcus* and *Rothia*. Given the previous reports of the potential role of *Streptococcus* in driving psoriasis [9, 10], it would be interesting to further investigate the role of *Rothia* spp. in psoriasis since it is highly co-abundant with *Streptococcus*. At the species level, *P*. *acnes*, which was more abundant in healthy skin, was negatively correlated with *S*. *sciuri* and *S*. *pettenkoferi*, both of which were enriched in the skin microbiota of psoriasis patients (Fig. 4b)*.* Consistent with this observation, we also found *P*. *acnes and S*. *epidermidis* to be significantly enriched in *S*. *aureus* low psoriasis samples and *S*. *pettenkoferi* was enriched in *S*. *aureus high* psoriasis samples (Additional file 1: Figure S3E), suggesting that the antagonistic interaction among these bacteria may contribute to pathogenesis. Interestingly, *Pseudoclavibacter bifida* was negatively correlated with *P*. *acnes* and positively correlated to *S*. *sciuri* (Fig. 4b). The abundance of *Pseudoclavibacter bifida* was also enriched in *S*. *aureus high* psoriasis samples (Additional file 1: Figure S3E). Moreover, *P*. *acnes* and *P*. *granulosum* serve as two predominant *Propionibacterium* species and our data shows that they are positively correlated with each other (Fig. 4b). The strong co-correlation of *P*. *acnes* and *P*. *granulosum* and their association to healthy skin suggests that these *Propionibacterium* spp. may have a role in maintaining skin health.

Psoriatic lesions are characterized by thick and highly inflamed skin plaques, so the psoriatic lesions, psoriasis non-lesional skin, and healthy skin represent very distinct microbial habitats that may affect the quality of interactions between different microbes. Consistent with this, we observed distinct species correlations in these disease states, supporting the hypothesis that different microbe-microbe interactions occur in each disease state. We found the most numerous and strongest microbe-microbe correlations in healthy skin samples (Fig. 4c). Surprisingly, species correlations in the microbial community associated with psoriatic lesions (Fig. 4e) were more similar to those in healthy skin than psoriatic non-lesional skin (Fig. 4d). In healthy skin, *P*. *acnes* was negatively correlated with several bacterial species (Fig. 4c), suggesting it may inhibit the growth of these bacteria. Fewer microbes were negatively correlated with *P*. *acnes* in psoriatic non-lesional skin (Fig. 4d) and only *Pseudoclavibacter bifida* was anti-correlated with *P*. *acnes* in lesional skin (Fig. 4e). Overall, our data suggests the possibility that *P*. *acnes* may have a role in influencing the skin microbial community by keeping the growth of some microbes under control and that perturbation of this balance in psoriatic skin could serve as a potential disease driver.

### *S*. *aureus* triggers Th17 immune response in a murine model

**Fig. 5:**
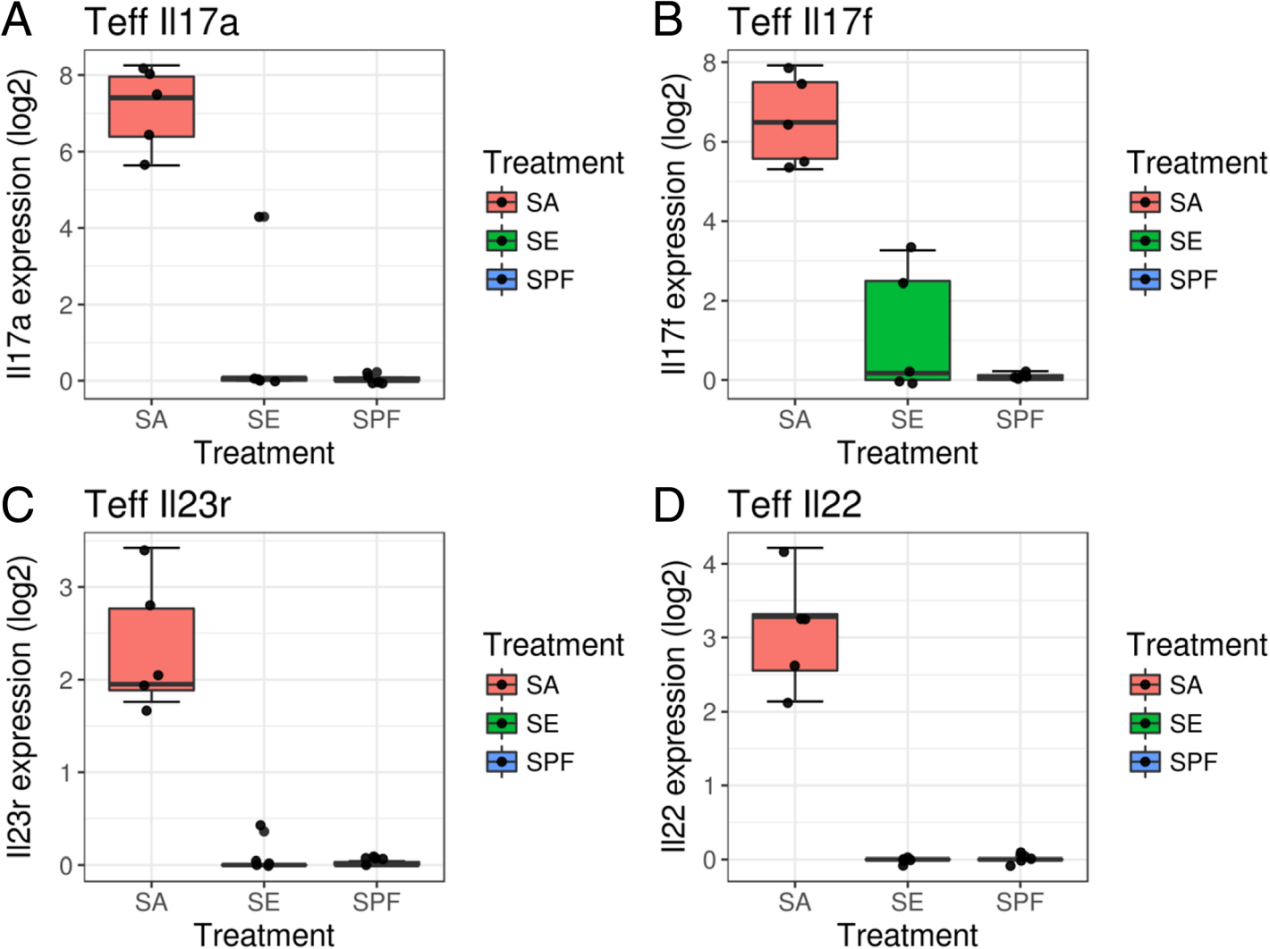
*Staphylococcus aureus* exposure triggers Th17 response in effector T cells. mRNA expression (log2FPKM) of cutaneous effector T cells from specific pathogen-free (SPF) mice colonized with *Staphylococcus aureus* (SA), *Staphylococcus epidermidis* (SE), or none (SPF). Compared to the SPF control, the *Stapylococcus aureus* colonization triggers gene expression in **a** IL-17A (adj *p* value = 3.51e−7), **b** IL-17F (adj *p* value = 3.08e−6), **c** IL-23R (adj *p* value = 3.74e−8), and **d** IL-22 (adj *p* value = 1.01e09). Colonization with *Staphylococcus epidermidis* does not trigger Th17 response

The increased prevalence of *S*. *aureus* in both lesional and non-lesional skin of psoriasis patients suggested the possibility that *S*. *aureus* might play a role in early stages of psoriasis pathogenesis. Despite its undesirable role in the context of psoriasis, the Th17 response serves as one of the major arms of host defense against bacterial infection through promotion of B cell activation and attraction of neutrophils [36, 37]. IL-17 is crucial in clearance of *S*. *aureus* at nasal, skin, and soft tissue sites [37]. Previous studies have shown that *S*. *aureus* proteins promote Th17 differentiation in vitro [38], suggesting that colonization by *S*. *aureus* can lead to increased Th17 activation and IL-17 secretion. To assess the effect of *S*. *aureus* colonization on Th17 response in the skin, we performed skin colonization of newborn-specific pathogen-free (SPF) mice with *S*. *aureus* strain USA300 and assessed the cutaneous effector CD4^+^ T (Teff) cell response using RNAseq in comparison with SPF mice colonized with the commensal *S*. *epidermidis*, or un-colonized SPF controls (Table 10). We found significantly stronger Th17 transcriptomic signals in Teff cells sorted from the skin *S*. *aureus*-colonized mice. Teff cells isolated from mice exposed to *S*. *aureus* expressed significantly higher levels of IL-17A and IL-17F cytokine transcripts (Fig. 5a, b). IL-17A has been well characterized as one of the major drivers for psoriasis pathogenesis whereas IL-17F shares some redundant functions to IL-17A but its role in psoriasis is less defined [39]. Besides IL-17, other components of Th17 responses including IL23R and IL22 were also increased upon *S*. *aureus* exposure (Fig. 5c, d). While *S*. *aureus* exposure during early life triggers a strong Th17 response in mice, the same treatment did not elicit consistent activation of a Th1 response (Additional file 1: Figure S4). Although *S*. *aureus* colonization has been strongly associated with atopic dermatitis, which is driven by Th2 responses [8, 40, 41], most components of the Th2 response such as IL-4, IL-5, and IL-13 were not induced by early colonization of *S*. *aureus* (Additional file 1: Figure S5). However, we did observe a strong induction in expression of the Th2-promoting transcription factor, GATA3 (Additional file 1: Figure S5E). Together, our data suggests that *S*. *aureus* colonization can specifically trigger activation of Th17 response, which might contribute to IL-17-driven inflammation in psoriasis.

**Table 10:** Genes differentially expressed in skin T effector cells of SPF + SA-colonized mice vs. SPF-colonized mice

Gene	Log2FoldChange	*p* value	*p* adj	absFC
Adgrl4	12.05	1.11E−21	7.51E−19	12.05
Efnb2	12.04	1.30E−20	7.82E−18	12.04
Ptprb	11.95	2.57E−16	5.87E−14	11.95
Sele	11.47	6.20E−10	2.94E−08	11.47
Procr	11.41	7.75E−12	6.08E−10	11.41
Il17a	11.38	5.36E−27	1.29E−23	11.38
Galnt15	11.37	7.57E−20	3.87E−17	11.37
Cyyr1	11.28	2.11E−18	7.90E−16	11.28
Rgs4	11.28	2.77E−20	1.51E−17	11.28
Flt1	11.27	4.15E−17	1.17E−14	11.27
Btnl9	11.24	1.83E−17	5.72E−15	11.24
Nts	11.24	1.35E−13	1.72E−11	11.24
Lamb2	11.07	2.31E−16	5.41E−14	11.07
Car4	11.00	1.67E−18	6.41E−16	11.00
Lss	10.97	5.12E−12	4.30E−10	10.97
Blk	10.97	1.17E−09	5.16E−08	10.97
RP24-360B3.1	10.84	1.60E−16	4.02E−14	10.84
Tinagl1	10.84	4.26E−19	1.85E−16	10.84
Stc1	10.80	1.80E−12	1.69E−10	10.80
Me1	10.78	2.90E−15	5.20E−13	10.78
Aqp7	10.73	2.58E−12	2.27E−10	10.73
Ptprm	10.69	1.62E−17	5.16E−15	10.69
Rasip1	10.62	1.87E−12	1.73E−10	10.62
Itga7	10.61	4.83E−15	8.40E−13	10.61
Rbp7	10.56	5.20E−09	1.99E−07	10.56
Selp	10.54	7.89E−12	6.13E−10	10.54
Il17f	10.53	2.25E−14	3.42E−12	10.53
Angpt2	10.50	2.37E−12	2.14E−10	10.50
Hid1	10.47	3.39E−17	1.00E−14	10.47
Adamts9	10.45	7.13E−16	1.43E−13	10.45
Sqle	10.44	2.08E−13	2.51E−11	10.44
Pdgfb	10.44	7.58E−12	5.98E−10	10.44
Gpihbp1	10.41	1.07E−16	2.77E−14	10.41
Galnt18	10.37	4.46E−17	1.23E−14	10.37
Sned1	10.36	3.86E−13	4.37E−11	10.36
Sulf1	10.32	6.17E−16	1.29E−13	10.32
Lamc3	10.30	1.44E−10	7.91E−09	10.30
Stap2	10.25	8.80E−19	3.62E−16	10.25
Ccm2l	10.22	2.11E−14	3.27E−12	10.22
Tnc	10.21	2.22E−12	2.03E−10	10.21
Esrp1	10.17	7.74E−10	3.56E−08	10.17
Cyp17a1	10.15	2.27E−08	7.07E−07	10.15
Nrip2	10.15	1.06E−16	2.77E−14	10.15
Alas2	10.11	4.62E−13	5.13E−11	10.11
Aadac	10.10	7.90E−08	2.09E−06	10.10
Unc45b	10.10	3.29E−11	2.14E−09	10.10
Enpp4	10.07	5.32E−13	5.72E−11	10.07
Pappa2	10.06	4.75E−12	4.05E−10	10.06
Tcrg-C4	10.04	6.93E−14	9.59E−12	10.04
Bcam	10.02	2.25E−13	2.67E−11	10.02
Tie1	10.01	7.30E−12	5.78E−10	10.01
Gja5	9.99	2.45E−10	1.28E−08	9.99
Rassf9	9.98	1.20E−14	1.97E−12	9.98
Tfap2c	9.98	6.73E−16	1.37E−13	9.98
Gdap10	9.97	1.67E−16	4.16E−14	9.97
Ppm1l	9.93	7.46E−14	1.02E−11	9.93
Ptch2	9.92	1.15E−12	1.15E−10	9.92
Vtn	9.91	6.14E−12	5.01E−10	9.91
Dll4	9.89	3.19E−14	4.67E−12	9.89
Fam73a	9.82	1.17E−13	1.53E−11	9.82
Pcdh17	9.82	2.72E−14	4.10E−12	9.82
Ptgs1	9.80	8.06E−12	6.24E−10	9.80
Slc12a1	9.79	1.88E−14	2.96E−12	9.79
Gm37297	9.77	4.01E−16	8.79E−14	9.77
Cyp1a1	9.74	5.32E−08	1.49E−06	9.74
Shroom4	9.73	1.35E−09	5.84E−08	9.73
Gm12158	9.72	1.18E−13	1.53E−11	9.72
Ackr3	9.71	2.01E−16	4.86E−14	9.71
Clstn3	9.67	7.71E−11	4.57E−09	9.67
Hmcn1	9.64	6.27E−16	1.29E−13	9.64
Tek	9.63	3.10E−19	1.46E−16	9.63
Esrp2	9.62	6.62E−11	3.98E−09	9.62
Ednra	9.61	4.06E−08	1.18E−06	9.61
Thsd7a	9.60	3.99E−10	2.01E−08	9.60
Tdrd9	9.59	1.23E−08	4.19E−07	9.59
Sfrp1	9.56	1.80E−10	9.60E−09	9.56
Aplnr	9.56	1.46E−17	4.83E−15	9.56
Mall	9.55	1.20E−14	1.97E−12	9.55
Mx2	9.55	2.10E−13	2.51E−11	9.55
Snca	9.55	7.99E−08	2.11E−06	9.55
Gm15740	9.55	4.67E−14	6.73E−12	9.55
Sectm1b	9.54	6.10E−15	1.05E−12	9.54
Tacr1	9.53	1.24E−07	3.06E−06	9.53
Rapgef5	9.52	1.49E−14	2.42E−12	9.52
Clec1a	9.52	5.45E−07	1.10E−05	9.52
Prox1	9.51	1.31E−09	5.71E−08	9.51
Clec14a	9.51	7.87E−08	2.09E−06	9.51
Rasgrf2	9.50	5.56E−12	4.62E−10	9.50
Dll1	9.50	1.93E−15	3.62E−13	9.50
Stac2	9.50	8.92E−12	6.70E−10	9.50
Celsr2	9.49	1.88E−11	1.27E−09	9.49
Robo1	9.48	1.30E−10	7.27E−09	9.48
Cxadr	9.47	7.82E−12	6.11E−10	9.47
Clic5	9.46	7.72E−09	2.82E−07	9.46
Taf9b	9.46	4.11E−14	5.98E−12	9.46
Spns2	9.46	2.74E−12	2.38E−10	9.46
Dhcr24	9.45	2.55E−13	2.94E−11	9.45
Gm37736	9.45	2.18E−14	3.34E−12	9.45
Gm16587	9.43	5.16E−07	1.05E−05	9.43
Ret	9.43	2.75E−08	8.36E−07	9.43
Tmem45b	9.43	1.37E−06	2.44E−05	9.43
Il22	9.42	1.82E−12	1.70E−10	9.42
Upp1	9.41	1.49E−13	1.88E−11	9.41
Paqr5	9.41	8.56E−10	3.84E−08	9.41
Piezo2	9.41	1.23E−10	6.96E−09	9.41
Psd2	9.40	5.60E−10	2.76E−08	9.40
Adtrp	9.40	4.97E−08	1.41E−06	9.40
Gk5	9.40	1.43E−07	3.46E−06	9.40
Cldn15	9.39	6.11E−11	3.73E−09	9.39
Aqp1	9.39	1.51E−13	1.90E−11	9.39
Heph	9.38	7.85E−13	8.13E−11	9.38
Emilin1	9.37	1.81E−15	3.44E−13	9.37
Plxna4	9.36	2.32E−09	9.56E−08	9.36
Ano1	9.36	7.20E−09	2.67E−07	9.36
Ebf3	9.36	2.28E−12	2.07E−10	9.36
Sgip1	9.35	3.58E−10	1.82E−08	9.35
Gm38125	9.34	4.44E−11	2.83E−09	9.34
Avpr1a	9.34	9.93E−08	2.55E−06	9.34
Tcrg-V6	9.34	7.86E−10	3.57E−08	9.34
Hoxd10	9.33	5.26E−14	7.46E−12	9.33
Dchs1	9.30	1.14E−13	1.51E−11	9.30
Hrct1	9.30	1.07E−08	3.74E−07	9.30
C1qtnf9	9.29	3.45E−17	1.00E−14	9.29
Lrg1	9.28	1.31E−15	2.55E−13	9.28
Fgfbp1	9.24	1.99E−07	4.60E−06	9.24
Eps8l2	9.24	5.95E−12	4.87E−10	9.24
RP24-188E19.4	9.22	6.55E−11	3.95E−09	9.22
Vsig10	9.21	4.56E−09	1.76E−07	9.21
Exd2	9.21	2.86E−14	4.28E−12	9.21
Rnd1	9.20	2.14E−10	1.13E−08	9.20
Adgrg6	9.19	4.55E−11	2.88E−09	9.19
Adcy4	9.18	1.40E−10	7.73E−09	9.18
Cyp2b19	9.18	2.24E−07	5.05E−06	9.18
Ndst3	9.15	1.94E−09	8.13E−08	9.15
Xlr4c	9.14	9.66E−13	9.82E−11	9.14
RP24-188E19.3	9.14	6.41E−13	6.76E−11	9.14
Stc2	9.14	2.37E−08	7.35E−07	9.14
Lmbr1	9.13	2.35E−07	5.24E−06	9.13
2610012C04Rik	9.13	3.39E−11	2.19E−09	9.13
Yes1	9.12	9.29E−09	3.32E−07	9.12
Sgcb	9.11	4.81E−08	1.37E−06	9.11
Vsig2	9.11	7.85E−08	2.09E−06	9.11
Zfpm2	9.10	9.96E−12	7.37E−10	9.10
Oaf	9.10	1.97E−07	4.55E−06	9.10
Sybu	9.09	1.23E−09	5.41E−08	9.09
Ebf2	9.08	2.05E−14	3.20E−12	9.08
Gm26667	9.08	1.06E−13	1.42E−11	9.08
Abca5	9.08	4.10E−08	1.19E−06	9.08
Lrat	9.07	9.53E−11	5.53E−09	9.07
Slc2a4	9.07	4.08E−09	1.59E−07	9.07
Zfp532	9.06	8.47E−12	6.47E−10	9.06
Grrp1	9.05	2.00E−11	1.34E−09	9.05
Slc2a13	9.04	5.70E−12	4.69E−10	9.04
Gm37783	9.02	1.95E−13	2.39E−11	9.02
Fat4	9.01	1.54E−09	6.59E−08	9.01
Slc33a1	9.00	1.77E−11	1.20E−09	9.00
Gtf2h2	9.00	1.32E−10	7.39E−09	9.00
C130079G13Rik	9.00	5.71E−06	8.18E−05	9.00
Col6a6	9.00	2.41E−12	2.16E−10	9.00
Adamtsl1	8.99	6.25E−07	1.23E−05	8.99
Pcdh12	8.98	2.24E−08	6.99E−07	8.98
Npy1r	8.98	1.53E−11	1.06E−09	8.98
Tfap2a	8.96	1.39E−06	2.47E−05	8.96
Gm38157	8.95	7.45E−10	3.47E−08	8.95
RP24-194 J1.1	8.95	4.42E−07	9.16E−06	8.95
Adgrf5	8.95	4.35E−24	4.89E−21	8.95
Sumf2	8.95	4.48E−08	1.28E−06	8.95
Moxd1	8.93	1.56E−10	8.50E−09	8.93
Fdxr	8.93	3.58E−09	1.41E−07	8.93
Colec11	8.92	6.02E−10	2.87E−08	8.92
St6galnac5	8.92	6.51E−10	3.06E−08	8.92
Pparg	8.91	3.72E−08	1.10E−06	8.91
Ndnf	8.90	5.09E−10	2.53E−08	8.90
Gm15712	8.90	3.20E−12	2.77E−10	8.90
Tcaf2	8.90	7.47E−08	2.00E−06	8.90
Adam12	8.90	1.98E−07	4.57E−06	8.90
Vstm4	8.89	1.24E−06	2.23E−05	8.89
Pkn3	8.88	2.55E−08	7.88E−07	8.88
RP23-363 M4.2	8.86	8.36E−12	6.41E−10	8.86
Neurl1b	8.84	5.73E−07	1.14E−05	8.84
4631405K08Rik	8.84	6.73E−13	7.01E−11	8.84
Dennd2c	8.83	1.57E−08	5.17E−07	8.83
Il23r	8.82	3.27E−12	2.82E−10	8.82
Cda	8.82	1.11E−08	3.84E−07	8.82
Higd1b	8.81	2.23E−06	3.63E−05	8.81
Plscr2	8.81	1.11E−06	2.03E−05	8.81
Lcn2	8.80	4.39E−10	2.21E−08	8.80
Lrrn1	8.80	5.24E−08	1.48E−06	8.80
Nipsnap1	8.78	3.79E−06	5.75E−05	8.78
Zfp57	8.77	9.44E−13	9.65E−11	8.77
Reep6	8.77	2.48E−07	5.51E−06	8.77
Dach1	8.76	5.34E−07	1.08E−05	8.76
Cpa6	8.76	1.47E−11	1.04E−09	8.76
Scube1	8.76	1.49E−07	3.58E−06	8.76
Tmem51	8.75	5.87E−10	2.82E−08	8.75
Prlr	8.74	5.71E−11	3.52E−09	8.74
Nova2	8.74	4.46E−07	9.23E−06	8.74
Arnt2	8.73	1.01E−07	2.59E−06	8.73
Cldn5	8.73	8.94E−12	6.70E−10	8.73
Slc6a17	8.73	4.66E−09	1.79E−07	8.73
Gucy1b3	8.73	1.90E−07	4.42E−06	8.73
Nrbp2	8.73	5.74E−06	8.20E−05	8.73
Fgfrl1	8.72	5.81E−10	2.80E−08	8.72
Fam13c	8.72	2.88E−06	4.53E−05	8.72
RP24-95O4.6	8.71	2.13E−06	3.50E−05	8.71
Apmap	8.71	9.28E−09	3.32E−07	8.71
Crygd	−8.70	3.17E−04	2.36E−03	8.70
Gdf10	8.70	1.43E−08	4.75E−07	8.70
Plxnb1	8.68	4.34E−08	1.25E−06	8.68
Foxc1	8.67	3.07E−11	2.00E−09	8.67
Has1	8.67	3.76E−19	1.67E−16	8.67
Hs3st1	8.67	8.06E−07	1.53E−05	8.67
Ppp1r3c	8.66	1.84E−07	4.33E−06	8.66
Dio2	8.66	7.06E−08	1.92E−06	8.66
Gjc3	8.66	1.04E−07	2.65E−06	8.66
Jmjd8	8.66	5.71E−10	2.80E−08	8.66
RP23-378O9.1	8.65	7.83E−09	2.85E−07	8.65
Zfp212	8.63	1.91E−07	4.45E−06	8.63
Acvr1	8.63	4.06E−07	8.51E−06	8.63
Zdhhc15	8.63	8.13E−10	3.68E−08	8.63
Lamb3	8.62	6.23E−09	2.34E−07	8.62
Slc26a7	8.62	5.24E−06	7.60E−05	8.62
Itgb4	8.62	7.00E−06	9.67E−05	8.62
Tmem56	8.62	1.33E−06	2.39E−05	8.62
Mtrr	8.62	9.62E−12	7.15E−10	8.62
Gm12122	8.62	3.62E−10	1.84E−08	8.62
Epas1	8.61	2.23E−17	6.86E−15	8.61
Vldlr	8.61	7.75E−07	1.48E−05	8.61
Ifitm5	8.61	2.02E−13	2.45E−11	8.61
Fam135a	8.61	3.93E−13	4.43E−11	8.61
Kcne4	8.60	1.98E−06	3.31E−05	8.60
Sort1	8.59	3.14E−07	6.84E−06	8.59
Mir1192	8.58	3.55E−10	1.81E−08	8.58
Pigl	8.58	7.32E−09	2.70E−07	8.58
Smoc1	8.58	1.09E−07	2.75E−06	8.58
Lrrc8b	8.58	7.78E−10	3.56E−08	8.58
Gm17096	8.58	7.74E−10	3.56E−08	8.58
Gm37524	8.58	2.42E−09	9.93E−08	8.58
Per3	8.57	9.01E−18	3.10E−15	8.57
Pvrl2	8.56	1.86E−07	4.36E−06	8.56
Adra2a	8.56	2.48E−08	7.66E−07	8.56
Plod2	8.56	2.98E−15	5.30E−13	8.56
Cryba4	8.55	3.26E−06	5.04E−05	8.55
RP24-360B3.3	8.55	1.33E−07	3.24E−06	8.55
Kcnj2	8.55	1.46E−07	3.52E−06	8.55
Tnfsf10	8.54	5.49E−09	2.09E−07	8.54
Pcdhga8_dup1	8.54	2.10E−07	4.79E−06	8.54
Ifi44	8.54	6.99E−06	9.67E−05	8.54
Adck1	8.53	6.01E−08	1.65E−06	8.53
Supv3l1	8.53	2.95E−09	1.18E−07	8.53
Pawr	8.53	6.18E−11	3.76E−09	8.53
Osmr	8.53	1.32E−08	4.43E−07	8.53
Derl3	8.52	8.50E−10	3.83E−08	8.52
RP24-188E19.2	8.50	1.62E−07	3.84E−06	8.50
Gm11730	8.50	6.43E−11	3.90E−09	8.50
B230216N24Rik	8.50	7.29E−09	2.69E−07	8.50
Pou2f3	8.49	2.87E−09	1.15E−07	8.49
Fam161b	8.49	1.10E−07	2.78E−06	8.49
Chchd10	8.49	6.25E−10	2.95E−08	8.49
Miat	8.48	2.21E−07	4.99E−06	8.48
Nr6a1	8.48	1.10E−08	3.82E−07	8.48
Clnk	8.48	3.53E−10	1.81E−08	8.48
RP24-303G10.1	8.48	2.05E−08	6.56E−07	8.48
Exoc3l2	8.48	1.41E−06	2.50E−05	8.48
Gm15609	8.47	5.80E−10	2.80E−08	8.47
Gm17477	8.47	6.01E−11	3.69E−09	8.47
Egfl8	8.47	1.56E−11	1.08E−09	8.47
Pcdhb17	8.47	1.01E−07	2.59E−06	8.47
Shb	8.47	2.08E−08	6.63E−07	8.47
Il7	8.46	8.04E−11	4.75E−09	8.46
Slc1a3	8.46	3.99E−06	6.04E−05	8.46
Ntrk3	8.46	4.16E−07	8.66E−06	8.46
Gypa	8.45	4.66E−06	6.85E−05	8.45
Tmem255b	8.45	2.10E−05	2.44E−04	8.45
Tgfa	8.44	5.81E−10	2.80E−08	8.44
6430590A07Rik	8.43	9.61E−10	4.28E−08	8.43
Ltbp2	8.43	2.02E−07	4.65E−06	8.43
Gm17491	8.43	3.53E−07	7.55E−06	8.43
4-Sep	8.43	1.00E−23	9.51E−21	8.43
Mboat2	8.43	1.44E−07	3.50E−06	8.43
Tle2	8.42	1.11E−08	3.84E−07	8.42
Gjb3	8.42	2.70E−05	3.01E−04	8.42
Rassf10	8.42	1.73E−08	5.62E−07	8.42
Dnm3os	8.42	2.43E−12	2.16E−10	8.42
Tenm4	8.42	6.98E−09	2.60E−07	8.42
D630008O14Rik	8.41	4.49E−06	6.65E−05	8.41
Enpp3	8.40	6.18E−09	2.32E−07	8.40
Kcna2	8.40	9.09E−07	1.71E−05	8.40
Gm15844	8.40	2.84E−05	3.12E−04	8.40
Tor4a	8.40	1.53E−06	2.67E−05	8.40
Trp63	8.39	1.37E−12	1.33E−10	8.39
Myo1d	8.39	1.02E−05	1.34E−04	8.39
Ctif	8.39	6.62E−12	5.34E−10	8.39
Calm4	8.39	1.18E−05	1.51E−04	8.39
Serpinb1c	8.39	8.28E−08	2.17E−06	8.39
RP24-421E18.7	8.38	6.27E−12	5.09E−10	8.38
Dgkh	8.38	8.78E−12	6.65E−10	8.38
Bnc2	8.38	3.63E−11	2.34E−09	8.38
Pfkfb2	8.38	1.28E−08	4.31E−07	8.38
Pcdh9	8.37	1.06E−07	2.69E−06	8.37
Abca12	8.37	1.07E−06	1.97E−05	8.37
Fzd4	8.37	8.16E−09	2.96E−07	8.37
Csrnp2	8.35	1.68E−08	5.49E−07	8.35
Cds1	8.35	2.64E−09	1.07E−07	8.35
Tnn	8.35	1.54E−05	1.89E−04	8.35
Kcna5	8.35	1.78E−09	7.56E−08	8.35
Fermt1	8.34	9.76E−07	1.82E−05	8.34
Comp	8.34	3.31E−08	9.89E−07	8.34
Pkp1	8.34	1.93E−09	8.11E−08	8.34
Hephl1	8.33	6.29E−06	8.88E−05	8.33
Nxpe4	8.33	2.67E−09	1.08E−07	8.33
Prkd1	8.33	1.70E−06	2.91E−05	8.33
Gm7162	8.33	3.33E−09	1.32E−07	8.33
Tfap2b	8.33	2.37E−07	5.28E−06	8.33
RP24-496C22.5	8.33	2.49E−09	1.01E−07	8.33
Ptprr	8.33	3.74E−05	3.95E−04	8.33
Cacna1c	8.33	9.05E−10	4.04E−08	8.33
Fam57b	8.32	3.82E−09	1.50E−07	8.32
RP24-360B3.2	8.32	1.88E−12	1.73E−10	8.32
Gtf2ird1	8.31	2.43E−11	1.62E−09	8.31
Tnfrsf22	8.31	2.04E−10	1.08E−08	8.31
P3h1	8.30	1.23E−07	3.03E−06	8.30
3110001I22Rik	8.30	5.23E−07	1.06E−05	8.30
Dmpk	8.30	5.10E−13	5.52E−11	8.30
Tmem41a	8.30	1.01E−11	7.44E−10	8.30
Hoxa5	8.30	2.15E−07	4.88E−06	8.30
Myocd	8.30	3.07E−08	9.24E−07	8.30
Ackr1	8.30	1.04E−11	7.65E−10	8.30
Sox6	8.29	1.95E−06	3.27E−05	8.29
Schip1_dup1	8.29	7.42E−08	1.99E−06	8.29
Tc2n	8.29	3.43E−09	1.36E−07	8.29
Ptpn14	8.29	5.58E−10	2.76E−08	8.29
Zglp1	8.29	9.16E−08	2.37E−06	8.29
Trmt11	8.29	1.62E−09	6.91E−08	8.29
C2cd2l	8.28	2.57E−08	7.91E−07	8.28
RP23-333I5.3	8.28	3.72E−06	5.67E−05	8.28
Pkhd1l1	8.28	1.13E−07	2.85E−06	8.28
Nxpe2	8.28	6.37E−07	1.25E−05	8.28
Tex15	8.28	6.48E−08	1.77E−06	8.28
Syt7	8.27	2.03E−08	6.49E−07	8.27
Mmgt2	8.26	1.85E−08	5.96E−07	8.26
AI838599	8.26	1.20E−07	2.97E−06	8.26
Prr9	8.26	2.11E−05	2.45E−04	8.26
Srd5a3	8.25	1.54E−09	6.59E−08	8.25
Slc35f1	8.25	5.82E−09	2.20E−07	8.25
Tmtc4	8.25	1.67E−05	2.01E−04	8.25
Cyp2e1	8.25	9.70E−07	1.81E−05	8.25
RP23-157G2.2	8.24	2.86E−08	8.66E−07	8.24
Plin4	8.23	4.95E−08	1.40E−06	8.23
Heatr5b	8.23	7.49E−09	2.76E−07	8.23
Lhx6	8.22	2.28E−05	2.62E−04	8.22
Ccdc85a	8.22	6.36E−08	1.74E−06	8.22
RP23-463H10.1	8.21	1.18E−06	2.15E−05	8.21
RP23-465A17.7	8.21	5.93E−08	1.64E−06	8.21
Pof1b	8.21	5.35E−06	7.75E−05	8.21
Vwa3a	8.21	1.65E−05	2.00E−04	8.21
Rhbdd2	8.20	6.07E−10	2.89E−08	8.20
Zfp94	8.19	2.52E−09	1.03E−07	8.19
Zp1	8.18	6.28E−07	1.23E−05	8.18
Gm38142	8.18	1.79E−08	5.80E−07	8.18
Fam174b	8.18	6.66E−12	5.35E−10	8.18
Gm37399	8.18	7.40E−07	1.42E−05	8.18
Lrig3	8.17	1.02E−08	3.60E−07	8.17
Tcea2	8.17	1.89E−06	3.18E−05	8.17
Gm20696	8.15	1.01E−05	1.32E−04	8.15
Sfxn4	8.15	5.76E−10	2.80E−08	8.15
Prom1	8.15	8.89E−06	1.19E−04	8.15
Has2	8.15	2.20E−08	6.92E−07	8.15
Mamstr	8.14	3.33E−08	9.93E−07	8.14
Cadps2	8.14	2.57E−07	5.68E−06	8.14
Fignl2	8.14	3.52E−09	1.39E−07	8.14
Il17rd	8.14	1.77E−10	9.49E−09	8.14
Susd4	8.14	7.03E−07	1.36E−05	8.14
Spock1	8.13	3.04E−06	4.75E−05	8.13
Ptger2	8.13	6.56E−07	1.28E−05	8.13
Nudt12	8.13	9.58E−07	1.79E−05	8.13
Frem2	8.13	1.84E−06	3.11E−05	8.13
Wfdc3	8.13	1.05E−05	1.37E−04	8.13
Gpr4	8.12	3.49E−05	3.72E−04	8.12
Akap6	8.12	2.24E−06	3.65E−05	8.12
Gprasp2	8.11	1.27E−08	4.30E−07	8.11
Sncaip	8.11	2.29E−05	2.63E−04	8.11
Prkab1	8.11	7.89E−24	8.32E−21	8.11
Gm37780	8.11	1.17E−05	1.50E−04	8.11
Unc5c	8.10	2.23E−07	5.03E−06	8.10
Gm37648	8.09	2.13E−06	3.50E−05	8.09
Gkn3	8.09	4.75E−05	4.83E−04	8.09
St6galnac2	8.09	4.03E−05	4.21E−04	8.09
Klhl23	8.08	2.90E−08	8.78E−07	8.08
Olfr78	8.07	3.25E−07	7.05E−06	8.07
RP24-247A21.1	8.07	2.02E−05	2.36E−04	8.07
Dos	8.07	1.49E−11	1.05E−09	8.07
Scn3a	8.07	6.89E−11	4.11E−09	8.07
Jade3	8.06	3.68E−06	5.62E−05	8.06
Fam110b	8.06	4.07E−06	6.12E−05	8.06
4930578C19Rik	8.06	4.64E−06	6.82E−05	8.06
Tmed8	8.06	1.87E−07	4.37E−06	8.06
Hdhd3	8.04	1.83E−09	7.76E−08	8.04
RP24-147H20.3	8.04	7.10E−05	6.78E−04	8.04
Adamts20	8.04	1.81E−05	2.15E−04	8.04
B3gnt3	8.04	4.03E−07	8.49E−06	8.04
Mal2	8.03	3.77E−05	3.98E−04	8.03
Tmem41b	8.03	1.44E−16	3.69E−14	8.03
Efhd1	8.03	1.47E−06	2.60E−05	8.03
Glce	8.02	2.49E−06	3.99E−05	8.02
Rragd	8.02	7.09E−06	9.80E−05	8.02
Vipr2	8.02	6.53E−06	9.15E−05	8.02
Htr7	8.02	8.62E−07	1.63E−05	8.02
Hbb-bt	8.02	2.48E−15	4.56E−13	8.02
Wipf3	8.01	1.35E−06	2.41E−05	8.01
Gm14085	8.01	3.05E−10	1.58E−08	8.01
AI846148	8.01	2.64E−06	4.18E−05	8.01
Unc13b	7.99	1.05E−07	2.68E−06	7.99
Gm37519	7.99	6.67E−09	2.49E−07	7.99
Dnah6	7.98	1.82E−06	3.09E−05	7.98
Zdbf2	7.97	3.06E−06	4.78E−05	7.97
Chodl	7.97	4.73E−05	4.81E−04	7.97
Tbc1d19	7.97	4.07E−06	6.12E−05	7.97
Aoc3	7.97	5.53E−07	1.11E−05	7.97
Lgr6	7.97	5.73E−06	8.18E−05	7.97
Prss36	7.96	3.98E−08	1.17E−06	7.96
Zcchc18	7.96	1.70E−05	2.04E−04	7.96
Ak4	7.95	3.57E−05	3.80E−04	7.95
Pde4c	7.95	1.04E−07	2.66E−06	7.95
Ring1	7.95	7.44E−10	3.47E−08	7.95
Kcnb1	7.95	1.15E−07	2.88E−06	7.95
Rergl	7.94	7.72E−05	7.24E−04	7.94
Ccdc67	7.94	3.42E−07	7.34E−06	7.94
Ptx3	7.94	2.26E−06	3.66E−05	7.94
Cc2d2a	7.93	4.84E−10	2.42E−08	7.93
Efcab7	7.93	9.20E−06	1.23E−04	7.93
Gm436	7.92	1.19E−04	1.03E−03	7.92
Srd5a1	7.92	6.83E−07	1.32E−05	7.92
Stac	7.92	3.77E−06	5.74E−05	7.92
Pbld2	7.92	5.53E−07	1.11E−05	7.92
Atoh8	7.91	1.55E−06	2.69E−05	7.91
Dhrs13	7.91	1.26E−08	4.27E−07	7.91
Hoxb7	7.91	1.20E−06	2.17E−05	7.91
Cox18	7.90	1.50E−10	8.21E−09	7.90
B430010I23Rik	7.90	5.76E−10	2.80E−08	7.90
Acaa1b	7.90	5.23E−07	1.06E−05	7.90
Micall2	7.90	3.92E−06	5.94E−05	7.90
Galns	7.89	2.87E−06	4.51E−05	7.89
RP24-496C22.2	7.89	8.04E−06	1.10E−04	7.89
Armcx5	7.88	7.69E−11	4.57E−09	7.88
Sox17	7.87	5.06E−07	1.04E−05	7.87
Tmem110	7.87	5.65E−14	7.88E−12	7.87
C130023A14Rik	7.87	2.61E−11	1.71E−09	7.87
RP23-293 K21.1	7.87	1.65E−06	2.84E−05	7.87
Cx3cl1	7.86	6.88E−08	1.87E−06	7.86
Atat1	7.86	5.25E−05	5.25E−04	7.86
Dsg2	7.86	3.85E−07	8.13E−06	7.86
Aldh1a7	7.86	1.04E−04	9.22E−04	7.86
Zfp69	7.85	2.09E−06	3.45E−05	7.85
Myh14	7.85	6.81E−07	1.32E−05	7.85
Afap1l1	7.85	3.18E−26	5.97E−23	7.85
Mpzl2	7.85	6.86E−06	9.54E−05	7.85
Flywch2	7.84	2.73E−08	8.32E−07	7.84
Krtcap3	7.84	2.53E−05	2.86E−04	7.84
Epb4.1l4b	7.83	1.30E−05	1.64E−04	7.83
Ficd	7.83	3.45E−06	5.31E−05	7.83
Sh3gl3	7.83	4.95E−06	7.21E−05	7.83
Cyb561	7.83	2.01E−06	3.35E−05	7.83
Gm7909	7.83	1.84E−07	4.31E−06	7.83
Erf	7.83	2.74E−08	8.34E−07	7.83
Scgb3a1	7.82	8.59E−07	1.62E−05	7.82
Cwh43	7.82	4.37E−08	1.25E−06	7.82
Tfap2e	7.82	5.73E−08	1.59E−06	7.82
Pou6f1	7.82	4.56E−06	6.72E−05	7.82
Plxna2	7.82	6.16E−20	3.25E−17	7.82
Fstl4	7.82	4.96E−08	1.40E−06	7.82
Lmln	7.81	4.34E−08	1.25E−06	7.81
RP24-560A18.1	7.81	3.62E−06	5.55E−05	7.81
Prkg1	7.81	4.28E−06	6.38E−05	7.81
RP24-399A15.2	7.80	2.45E−05	2.79E−04	7.80
Cspg4	7.80	1.05E−12	1.06E−10	7.80
Tmem86a	7.80	1.88E−05	2.22E−04	7.80
Tll1	7.79	5.08E−05	5.11E−04	7.79
Laptm4b	7.79	7.70E−09	2.82E−07	7.79
6430573F11Rik	7.79	5.68E−07	1.13E−05	7.79
Gm26603	7.79	1.02E−07	2.61E−06	7.79
Ptk7	7.78	8.48E−08	2.22E−06	7.78
Igsf9	7.78	8.54E−06	1.15E−04	7.78
RP23-372C7.4	7.78	2.73E−05	3.04E−04	7.78
Nol4l	7.78	4.88E−08	1.39E−06	7.78
Slc7a2	7.78	1.20E−11	8.69E−10	7.78
Zfp52	7.77	2.22E−05	2.56E−04	7.77
A730049H05Rik	7.77	2.35E−05	2.69E−04	7.77
Yars2	7.77	8.91E−08	2.32E−06	7.77
Mc5r	7.77	6.54E−05	6.31E−04	7.77
Gm20699	7.76	1.54E−08	5.07E−07	7.76
4933407K13Rik	7.76	1.15E−04	1.00E−03	7.76
Tbc1d8	7.76	2.86E−18	1.03E−15	7.76
Gm9917	7.75	7.64E−09	2.80E−07	7.75
Aplp1	7.75	5.73E−06	8.18E−05	7.75
4933416E03Rik	−7.75	7.80E−04	4.99E−03	7.75
Nfatc4	7.75	2.59E−11	1.70E−09	7.75
Cpeb1	7.74	2.24E−08	6.99E−07	7.74
Bahcc1	7.74	8.17E−07	1.55E−05	7.74
Scarb1	7.74	3.98E−17	1.14E−14	7.74

## Discussion

In this study, we profiled the skin microbiota of psoriasis patients and healthy controls using the NIH standardized protocol and with higher sequencing depth to gain a more comprehensive understanding in psoriasis-associated microbiome. Our data demonstrate that the psoriasis skin microbiome is more heterogeneous compared to that of healthy skin. The compositional variance in the psoriatic skin community could be attributable to local environmental changes that accompany or immediately precede psoriatic disease. Proliferating keratinocytes in psoriasis patients are a rich source of antimicrobial peptides such as LL37, β-defensin, and psoriasin [42]. The constant presence of these antimicrobial peptides could undermine equilibrium of the skin microbiome community and select for microbial species resistant to these antimicrobials. Based on our data, we speculate that a healthy skin microbial community consists of key stabilizer species, which may prevent growth of other species in the local microenvironment. In psoriatic skin, these stabilizer species may be outcompeted by invading pathogenic species and/or inhibited by chronic exposure to antimicrobial peptides, enabling colonization by pathogenic bacteria normally excluded from this niche. This could explain the higher heterogeneity that we observed in psoriatic skin. In contrast to our result, Alekseyenko et al. and Gao et al. observed decreased bacterial diversity in psoriatic skin compared with healthy skin [12, 14] while Fahlen et al. found no difference [13]. Consistent with all the previous studies, we observed a decrease in relative abundance of *Actinobacter* associated with psoriasis skin [12–14]. Similar to Fahlen et al., we observed overrepresentation of *Proteobacteria* in psoriasis skin while both Gao et al. and Alekseyenko et al. showed a reduced abundance of *Proteobacteria* in psoriasis skin. These discrepancies might be due to the inherent heterogeneity in microbiota composition observed on the skin of psoriatic patients or to different experimental designs. This highlights the need to use standardized protocols among different studies to enhance reproducibility and to allow for meta-analysis of study cohorts. It is important to note that all studies mentioned above including ours profile the skin microbial community using an OTU (operational taxonomic unit) approach which groups reads from part of the 16S rRNA gene in order to account for artifact variance introduced by sequencing error. The major limitation of this approach is that by grouping different sequence variants, subtle inter-species variance can be sacrificed, which can reduce the resolution of taxonomical assignment at the species level [43]. Despite the inherent limitation of OTU-based profiling, we were still able to gain species insights from our dataset.

Increased colonization of *S*. *aureus* in psoriatic skin has been reported previously in several small studies, but only a few of these examined unaffected skin from psoriasis patients [44]. Our data revealed a significant increase of colonization in both psoriatic lesional and non-lesional sites compared to the baseline levels of *S*. *aureus* colonization found in healthy skin. This suggests that the increase in *S*. *aureus* is less likely a consequence of structural change in the skin from psoriasis but rather might be an important factor in initiating disease. Indeed, the potential role of bacterial infection in initiating and exacerbating psoriasis has been shown in *Streptococcus* infection [9, 10]. Our data from a murine model of *Staphylococcal* skin colonization suggests that cutaneous exposure to *S*. *aureus* triggers a strong Th17-type response in skin Teff cells as evident by induction of IL-17A, IL-17F, and IL22 cytokines. IL22 not only triggers a pro-inflammatory response, it also inhibits terminal differentiation of keratinocytes which is one of the characteristics of psoriasis. This suggests a potential capability of *S*. *aureus* to initiate psoriasis through upregulating a Th17 response. It is important to note that *S*. *aureus* consists of many strains and some strains are more virulent than others depending on their expression on a variety of different toxins and other molecules. Our murine experiments utilized *S*. *aureus* strain SF8300 from the USA300 lineage, which contains Panton-Valentine leukocidin (PVL) and phenol-soluble modulins (PSMs) contributing to its virulence in skin and soft tissue infections. Colonization of *S*. *aureus* has long been implicated in the pathogenesis of atopic dermatitis [8, 45]. Consistent with a recent study demonstrating that the capacity to induce Th2-type inflammation is limited to specific *S*. *aureus* strains isolated from severe atopic dermatitis patients, we observed little induction in components of Th2 response except for GATA3, which is the transcription factor that is required for Th2 polarization [40]. Compared to the baseline level of *S*. *aureus* colonization in healthy controls, we found that *S*. *aureus* levels in some of the psoriasis samples were increased up to 90-fold. The increased *S*. *aureus* colonization in psoriasis could be due to the expansion of *S*. *aureus* strains seen in healthy controls or colonization with new *S*. *aureus* strains. However, due to lack of strain resolution of 16S rRNA-based profiling, we are not able to distinguish these two possibilities. In addition to strain-specific immunomodulating effects, our observation might suggest a temporal relationship between Th17 and Th2 polarization in response to *S*. *aureus* colonization and/or during neonatal development, as has been reported in murine models of atopic dermatitis [46]. Future studies examining the *S*. *aureus* strain diversity in psoriasis skin and skin immune response to *S*. *aureus* strains specifically isolated from patients with psoriasis would be of significant interest in further dissecting the role of this bacterium in psoriasis disease pathogenesis.

*Propionibacterium* is one of the most dominant skin commensal bacteria [24]. *P*. *acnes* has long been linked to acne vulgaris [47, 48], but recent studies suggest that *P*. *acnes* is also highly abundant in healthy skin and specific pathogenic *P*. *acnes* strains is one of the key determinants for acne vulgaris [49–51]. In this study, we found *Propionibacterium* to be abundant in healthy, lesional, and non-lesional skin but with highest abundance in healthy skin. Moreover, *P*. *acnes* and *P*. *granulosum* were two of the strongest microbial species associated with healthy skin. A possible explanation for the reduced abundance in *Propionibacterium* species in psoriatic lesions might be that reduced sebaceous content in psoriatic plaques contributes to a less favorable environment for *Propionibacterium* growth. The potential consequence of this reduction in *Propionibacterium* species in psoriasis is less clear. On one hand, *P*. *acnes* is known to produce propionate, a short chain fatty acid which can promote regulatory T cell in the colon [52], as well as RoxP (radical oxygenase of *Propionibacterium acnes*), which can potentially reduce oxidative stress and prevent skin inflammation [53]. In contrast, certain strains of *P*. *acnes* isolated from acne patients have the potential to induce higher IL-17 production compared to strains isolated from healthy subjects based on unknown mechanisms [54]. Although our current study does not have the resolution for *P*. *acnes* strain identification, identifying psoriasis-specific *P*. *acnes* isolates and compare them to those in healthy subject and assessing their differential genomic content and ability to modulate host T cell responses will be crucial in understanding whether the abundance or type of *P*. *acnes* in psoriasis patients contributes to their propensity for disease.

The association between *Staphylococcus sciuri* and psoriasis non-lesional skin is rather surprising since *S*. *sciuri* is better known as an animal-associated bacteria [55, 56]. *S*. *sciuri* has also been found in the human skin in both healthy and hospitalized individuals [57]. The clinical relevance of *S*. *sciuri* has become important since several studies have isolated *S*. *sciuri* from hospitalized patients and methicillin-resistant strains of *S*. *sciuri* can be a health hazard for hospitalized patients [58–62]. Our study provides the first observation of *S*. *sciuri* in the context of psoriasis. It is possible that the *S*. *sciuri* carriers of our cohort obtained the bacteria from a previous hospital visit since *S*. *sciuri* has been found to be persistently present in the hospital environment [63]. While the possible role of *S*. *sciuri* in psoriasis is unclear, we have observed an interesting pattern of *S*. *sciuri* in our cohort. Our data show that an increase in *S*. *sciuri* abundance is exclusively associated with psoriasis skin, particularly in non-lesional skin (Fig. 3c). Moreover, *S*. *sciuri* abundance is negatively correlated with *P*. *acnes*, which is highly enriched in healthy skin (Fig. 4b). Together, our results suggest *S*. *sciuri* may have a potential role in psoriasis pathogenesis.

In order to understand the roles of microbiome in human health, it is important to consider the microbial community as whole. Bacterial interactions are as important as the host environment in shaping the skin microbial community. The microbe-microbe relationship can be competitive or symbiotic. We performed a correlation analysis on the most abundant microbial genera and species to elucidate possible microbe-microbe interactions in healthy and psoriasis skin. We found an anti-correlation between *S*. *sciuri* and *P*. *acnes*, consistent to their respective disease state associations. To our surprise, although *S*. *aureus* is enriched in psoriatic skin and is known from other studies to have a competitive relationship with *S*. *epidermidis* and *P*. *acnes*, our data did not corroborate these negative associations. Possible explanations for this might include strain-specific interactions as well as the impact of the skin environment on inter-species interactions, the latter being supported by our finding that inter-bacterial correlation clusters differed by different disease state. It is important to note that microbe-microbe relationships suggested in our study are only correlative, further experimentation on isolated microbes will be needed to validate these relationships. Nonetheless, our work predicts strong candidates for the microbe-microbe relationships that may be crucial for psoriasis pathogenesis. Taken together, our correlation analysis demonstrates the highly complex relationship among skin bacteria by showing that these inter-microbial relationships are altered in psoriasis, possibly due to changes in the biochemical changes in skin environment and/or ecological pressure imposed by an altered host immune response.

## Conclusion

In this study, we adhered to a stringent sampling protocol and measured skin microbiome profiles associated with psoriasis skin at six different skin sites. Our data revealed higher diversity and heterogeneity in psoriatic skin relative to healthy skin. Taxonomic analyses revealed specific microbial signatures associated with each disease state at the genus and species levels. Intriguingly, we found *Staphylococcus aureus* to be more abundant in both psoriatic non-lesional and lesional skin while *Staphylococcus epidermidis*, *Propionibacterium acnes*, and *Propionibacterium granulosum* were more abundant in healthy skin. We further tested the impact of *Staphylococcus aureus* colonization on host response in murine skin and validated its capacity for Th17 polarization. Finally, we demonstrated that disease state can alter microbe-microbe interactions and co-associations possibly due to differences in the physical and chemical environment of the skin. Our study confirms that psoriasis is accompanied by a shift in the skin-resident microbial community and raises intriguing possibilities worthy of further exploration for how this might directly impact the host immune response and psoriasis pathogenesis.

## Methods

### Study cohort

Twenty-eight adult psoriasis patients and 26 healthy volunteers recruited from the San Francisco Bay area were enrolled in the study after providing informed consent. Individuals with abnormal coagulation studies, positive HIV screening test, or a known history of bleeding disorders, abdominal surgery, gastrointestinal cancer, inflammatory bowel disease, AIDS, or other immunosuppressive condition, or concurrent inflammatory skin condition were also excluded. All psoriasis patients had a diagnosis of psoriasis from a physician for at least 6 months prior to study enrollment, which was verified by study staff. To assess the psoriatic microbiome in an untreated state, subjects were excluded if they had received systemic biologic therapy in the last 6 months, non-biologic systemic medications (methotrexate, cyclosporine, corticosteroids, cyclophosphamide, retinoids, photochemotherapy) or antibiotics in the last month, or phototherapy or topical therapy in the last 2 weeks prior to skin swabbing. Healthy volunteers had no personal or family history of psoriasis.

### Specimen collection

Skin swabs and stool samples were collected according to the protocol outlined in the Manual of Procedures for the NIH Human Microbiome Project [19]. Study participants were asked to refrain from showering and using any substances on their skin (lotion, perfume, make-up, etc.) for at least 24 h prior to skin swabbing. Samples of the skin microbiome were collected using individually packed, sterile cotton swabs (Epicentre Catch-All Swabs). For each subject, the skin was swabbed at six standardized sites: scalp, trunk, axilla, arm, leg, and gluteal fold for healthy samples and psoriasis unaffected samples. Psoriatic lesional samples were only taken when psoriasis plaques were present at one of the six sites. Negative controls were obtained by exposing swabs to room air for 10 s. All samples were stored in − 80 °C while they awaited further processing.

### DNA sequencing

DNA was extracted from the skin swab samples using the MasterPure Yeast DNA Purification Kit (Epicentre) with bead beating method to lyse the bacterial cells. To prepare skin microbiome library for sequencing, 16S rRNA were amplified at V1 to V3 hypervariable region using a universal forward primer (V1_27F primer): 5′-AGAGTTTGATCCTGGCTCAG-3′ attached to 5′ Illumina adapter and indexed reverse primer (V3_534R primer): 5′-ATTACCGCGGCTGCTGG-3′ attached with 3′ Illumina adapter sequence. Amplicon PCR reactions were completed as follows (per reaction): 2 μl of gDNA, 1× final concentration of 10× LA PCR Buffer ll (Mg2 + free) (Takara Bio USA), 0.4 mM dNTPs, 0.4 uM forward and reverse primers, 1.25 U of TaKaRa LA Taq polymerase high fidelity (Takara Bio USA), and nuclease-free water to bring the final volume to 25 μl. PCR cycling protocol consisted of an initial denaturation of 5 min at 95 °C, 30 cycles of 30 s at 95 °C, and 30 s at 56 °C followed by 5 min at 72 °C. PCR reactions were subsequently cleaned up using Agencourt AMPure XP kit (Beckman Coulter), and the purified amplicons were quantified using Quant-iT PicoGreen dsDNA Assay Kit (Invitrogen). Samples with amplicon concentrations less than three times above the average air control amplicon concentrations were excluded from the subsequent sequencing. In average, 60–70 samples were pooled in equal molar quantities and the pooled library was purified using minElute PCR purification kit (Qiagen) for the final purification. The pooled library was sequenced on a Miseq sequencer (Illumina) as described in commercially provided protocol with 25% phiX DNA added as spike-in. Miseq reagent kit V3 (Illumina) was used to generate paired-end 300-bp reads. To avoid confounding from batch effects or possible external contamination that could affect our comparison between psoriasis and healthy skin microbiome, each Miseq sequencing run was balanced with samples from three or four healthy subjects and four psoriasis samples.

### Data process and OTU picking

After quality check, the high-quality pair-end reads were assembled into ~ 550-bp fragments using FLASH [64] and we performed the subsequent analysis using Qiime scripts [65]. The pair-end-assembled sequence fragments were first aligned against the Qiime supplied reference database for picking close OTUs. Subsequently, the unaligned sequences clustered into 97% identity operational taxonomical units (OTUs) using UCLUST [66]. A representative sequence from each OTU cluster was aligned against the GreenGenes core set alignment template using PyNAST [67]. The chimera due to the PCR errors were identified by ChimeraSlayer and excluded from the subsequent analyses [68]. The remaining chimera-free OTUs were then used to approximate the phylogenetic tree using FastTree [69]. We removed samples with less than 10,000 sequences to ensure adequate sample depth after rarefaction. The chimera-free sequences were rarefied into 11,766 per samples using the custom script analyses (https://github.com/alifar76/MicroNorm) to account for library size differences between samples.

### Subsequent analysis for community diversities, microbial signatures, and inter-microbial correlations

The rarefied OTU table was used for all the subsequent analyses using Qiime 1.8.0 [65] and R [70]. Four alpha diversity metrics—chao1 index, observed OTUs, Shannon’s diversity index, and Simpson’s diversity index—were calculated for samples in each disease state, and the significance between different disease states were evaluated by a linear mixed effect model to account for multiple sampling from the same patient. The linear mixed effect model was performed by using R package lmerTest(v.3.0.1) [71]. The R package Kendall (v.2.2) [72] was used to perform the Mann-Kendall trend test to detect significant trends of alpha diversity across different disease states.

Weighted UniFrac dissimilarity matrices were calculated to determine beta diversity using Qiime script. To account for multiple sampling of different body sites from the same subject, we applied linear mixed effects model to the first principal component coordinate from weighted UniFrac (scripts available in Additional file 2). The heterogeneity of microbial communities within each disease state were determined by the average weighted UniFrac distances between site-matched samples within each disease state, and the significance was determined by one-way analysis of variance (ANOVA) followed by Tukey’s multiple comparisons test done by GraphPad Prism 7 (statistics output in Additional file 3). The relative abundance of each microbial community was summarized by Qiime script at the phylum and genus level. The microbial signatures associated to each disease state at both genus and species level were identified by Lefse, which combines non-parametric Kruskal-Wallis test and linear discriminant analysis (LDA) [33]. Taxonomical features with *p* value < 0.05 and LDA effect size > 2.0 were considered significant microbial signatures. To evaluate the skin site- and skin type-specific diversity changes and microbial signatures, the OTU tables were further subsetted into different skin sites or different skin types. The diversity and marker analyses were performed as described above. We further investigated the difference between psoriasis samples with high *S*. *aureus* abundance and those with low *S*. *aureus* abundance seen in healthy skin. We defined the highest *S*. *aureus* abundance in healthy skin as baseline. Psoriasis samples (both lesional and non-lesional) with higher *S*. *aureus* abundance than the baseline level were considered “*S*. *aureus* high” and psoriasis samples with lower or equal *S*. *aureus* abundance as the baseline level were considered “*S. aureus* low”. Lefse analysis was performed to identify bacterial signatures associated with *S*. *aureus* high and *S. aureus* low samples. Lastly, the rarefied OTU table was summarized to genus (L6) and species (L7) by Qiime script and Spearman correlation was calculated using a R package, Hmisc (v. 4.1.1) [73], for the top 25 most abundant genera and top 30 most abundant species (scripts available in Additional file 4). The species-species correlations were further investigated within different disease states (scripts available in Additional file 4). Summarized taxonomic tables used in correlation analyses were available in Additional files 5 and 6.

### Mouse skin bacterial colonization

C57BL/6 mice were purchased from Jackson Laboratories (Bar Harbor, ME) then bred and maintained in the UCSF-specific pathogen-free facility. All animal experiments were performed in accordance with the NIH Guide for the Care and Use of Laboratory Animals and the guidelines of the Laboratory Animal Resource Center and Institutional Animal Care and Use Committee of the University of California, San Francisco.

Bacterial strains *Staphylococcus epidermidis* Tü3298 and *Staphylococcus aureus* SF8300 were grown in tryptic soy broth for 24–48 h; pelleted and cellular mass from 2.5 ml of saturated culture was re-suspended in 100 μl of PBS for colonization of each animal. Newborn C57BL6 mice were colonized starting on day 3 of life and every other day thereafter until post-natal day 19 by pipetting 100 μl of bacterial suspension onto their skin and distributing evenly using a sterile PBS-soaked cotton-tipped swab.

### Isolation and RNA sequencing of CD4+ effector T (Teff) and regulatory T (Treg) cells from skin of bacterially associated mice

To isolate skin T cells for staining and FACS sorting, mice were sacrificed at 21 days of age and the entire trunk skin harvested and lightly defatted. The skin was then minced with scissors and re-suspended in a 50 ml conical with 1–2 ml of digestion media comprised of 2 mg/ml collagenase XI, 0.5 mg/ml hyaluronidase, and 0.1 mg/ml DNase in RPMI with 1% HEPES, 1% penicillin-streptomycin, and 10% fetal calf serum. This mixture was incubated in a shaking incubated at 37 °C at 250 rpm for 45 min. An additional 15 ml of RPMI/HEPES/P-S/FCS media was then added, and the 50 ml conical was shaken vigorously by hand for 30 s. Another 15 ml of media was added, and then, the entire suspension was filtered through a sterile 100-mm cell strainer followed by a 40-mm cell strainer into a new 50 ml conical. The suspension was then pelleted, and the cell pellet was re-suspended in sort buffer (RPMI, 2 mM EDTA, 25 mM HEPES, 2% FBS) with U/ml RiboLock RNase inhibitor (Thermo Scientific) for staining for 30 min at 4 °C with fluorophore-conjugated antibodies specific for CD3, CD4, CD8, CD25, CD45, ICOS, TCRβ, and Tonbo Live-dead Ghost Dye. Teff (Live, CD45+, CD3int, CD4+, CD8neg, TCRβ+ CD25neg, ICOSneg) cells were then isolated via cell sorting on a MoFlo XDP (Beckman Coulter) in the UCSF Flow Cytometry Core. Cells were pelleted and flash frozen. RNA isolation was performed by Expression Analysis Q2 Solutions using QIAGEN RNeasy Spin columns and was quantified via Nanodrop ND-8000 spectrophotometer. RNA quality was checked by Agilent Bioanalyzer Pico Chip. The SMARTer Ultra Low input kit was used to generate cDNA libraries which were then sequenced to a 25-M read depth with Illumina RNASeq.

Reads were aligned to UCSC GRCm38/mm10 reference genome with STAR software (2.4.2a) [70]. SAM files were generated with SAMtools from alignment results [71]. Read counts were obtained with htseq-count (0.6.1p1) with the union option [72]. Differential expression was determined using the R/Bioconducter package DESeq2 [73]. RPKM table was generated by Cuffdiff Cuffdiff (2.2.1) [74] (Additional file 7).

## Additional files

Additional file 1: **Figure S1**. Box plot shows the average weighted UniFrac distances among samples within each disease state at (a) arm, (b)trunk, (c)leg, (d)axilla, (e)gluteal fold and (f)scalp (*: *p*-value < 0.05, **: *p*-value < 0.01***: *p*-value < 0.001****: *p*-value < 0.0001). **Figure S2**. Box plot shows the average weighted UniFrac distances among samples within each disease state in (a) dry and (b)moist skin group (*: *p*-value < 0.05, **: *p*-value < 0.01***: *p*-value < 0.001****: *p*-value < 0.0001). **Figure S3**. Relative abundance of P. acnes and S. aureus in each disease state at different body sites. Relative abundance of P. acnes in healthy (red), psoriasis lesional (green), psoriasis non-lesional (blue) skin at (a) different skin sites and (b) different skin types (*: p-val < 0.05, **: p-val < 0.01, ****: p-val < 0.0001). (c) Box plot showing S. aureus abundance in S. aureus high samples. S. aureus high samples were defined as samples with higher S. aureus abundance than the highest S. aureus abundance among the healthy samples (baseline = 0.0068). (d) Bar graph depicts the prevalence of S. aureus high samples at each skin site in psoriasis lesional (blue bars) and psoriasis non-lesional (orange bars) skin. (e) Bacterial species associated with S. aureus high samples (red bars) and S. aureus low samples (green bars). **Figure S4**. Expression of Th1 components in effector T cells in response to Staphylococcus aureus colonization. The expression of Th1 components (a) T-bet (b) IFNγ and (c)IL-2 are comparable in all experimental groups. **Figure S5**. Expression of Th2 components in effector T cells in response to Staphylococcus aureus colonization. The expression of Th2 cytokines (a) IL-4 (b) IL-5, (c) IL-13 (d) IL-9 are comparable in all experimental groups. The expression of Th2 promoting transcription factor (e) GATA3 is induced by early colonization of S. aureus (adj. *p*-value = 1.49e-16); whereas, another Th2 transcription factor (f) STAT6 is not significantly induced. (PDF 405 kb)

Additional file 2: R notebook for applying linear mixed effects model to test the significance of difference in microbial diversity among psoriatic lesional, psoriatic unaffected, and healthy skin. (HTML 772 kb)

Additional file 3: Output of GraphPad Prism for statistics of heterogeneity of microbial communities isolated from skin associated with different disease state. (XML 18 kb)

Additional file 4: R notebook for microbe-microbe correlation analyses at genus and species level. (HTML 1518 kb)

Additional file 5: OTU table summarized to genus level used for correlation analysis. (CSV 1031 kb)

Additional file 6: OTU table summarized to species level used for correlation analysis. (CSV 295 kb)

Additional file 7: Mann-Kendall Test to detect trend in alpha diversity. (HTML 785 kb)
