# Supplementary material for: Alteration of the cutaneous microbiome in psoriasis and potential role in Th17 polarization
Source: Microbiome. 2018 Sep 5;6:154. doi: 10.1186/s40168-018-0533-1 (PMC6125946; doi:10.1186/s40168-018-0533-1)

Supplementary Figure 1

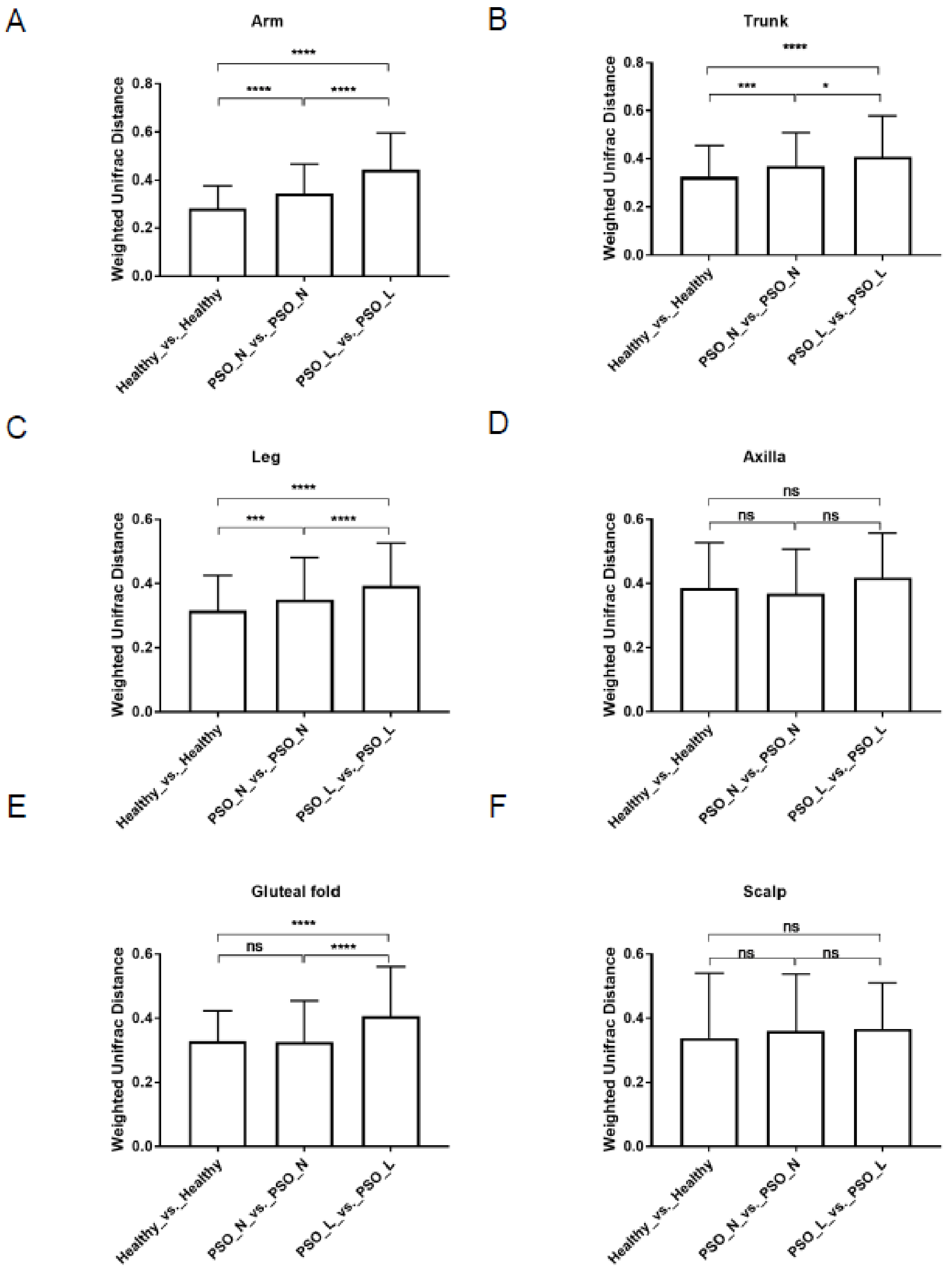

Supplementary Figure 2

A

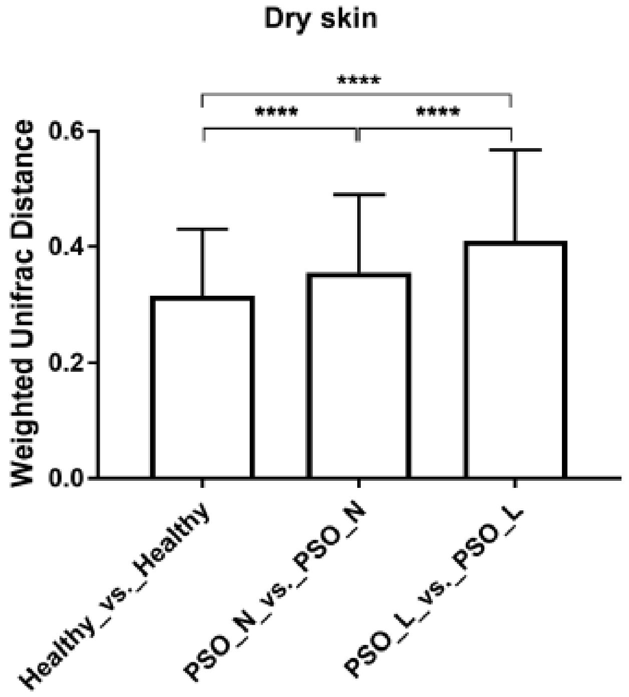

B

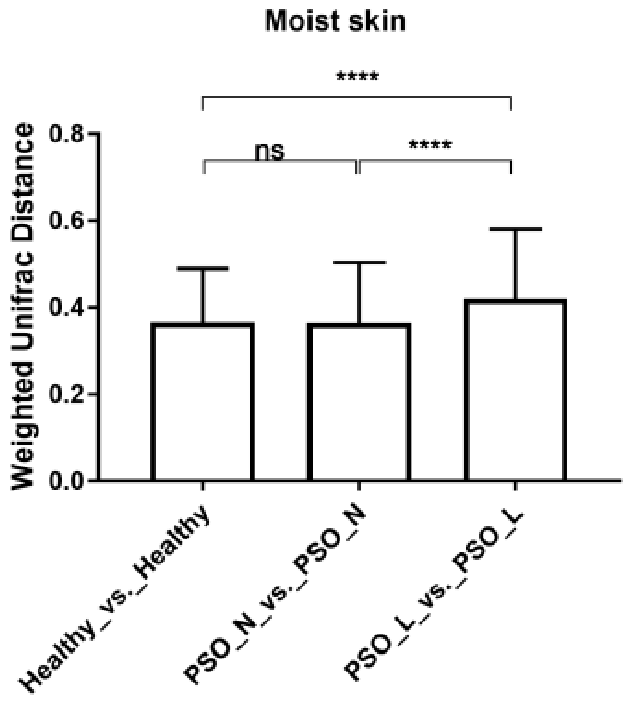

Supplementary Figure 3:

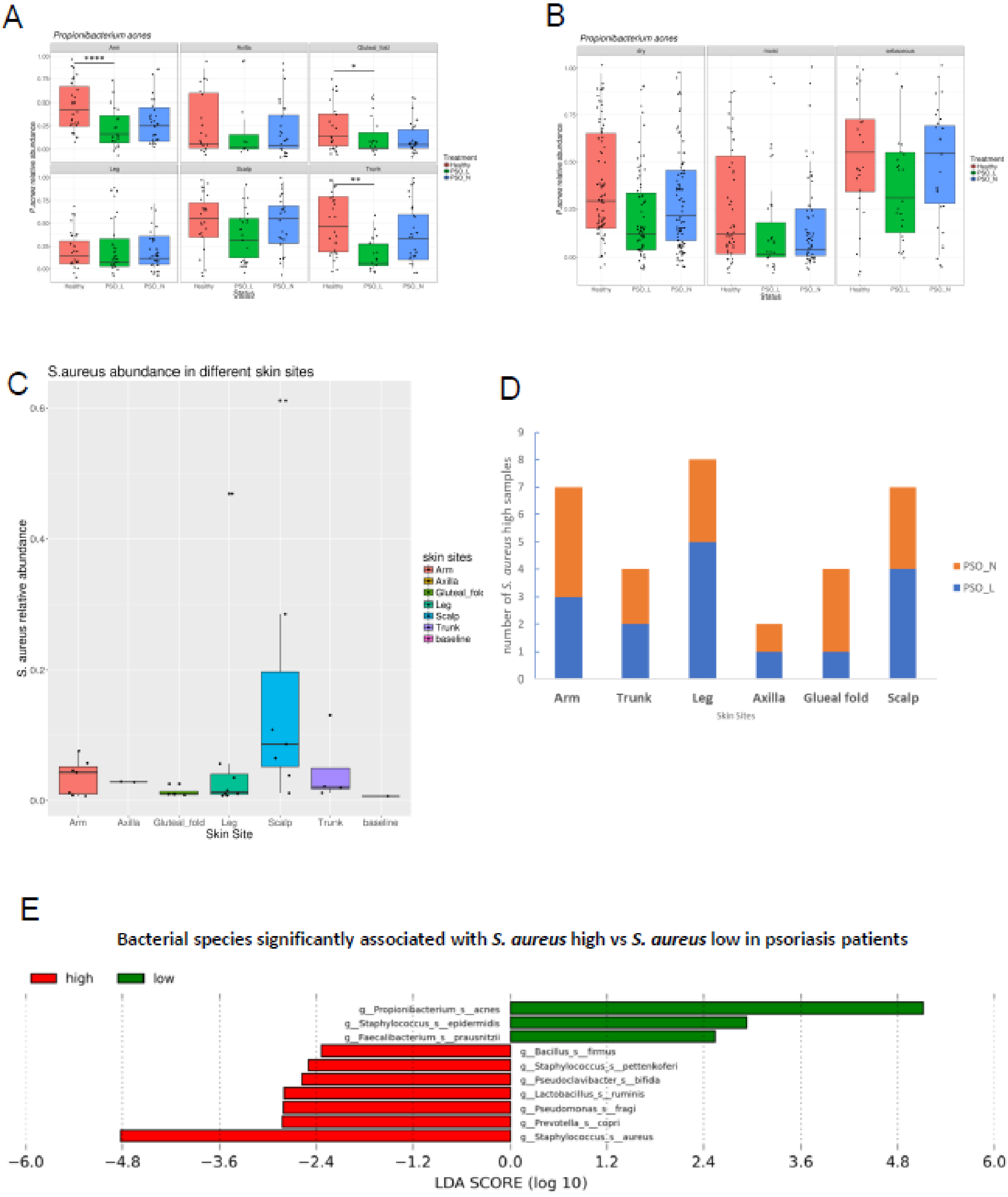

Supplementary Figure 4

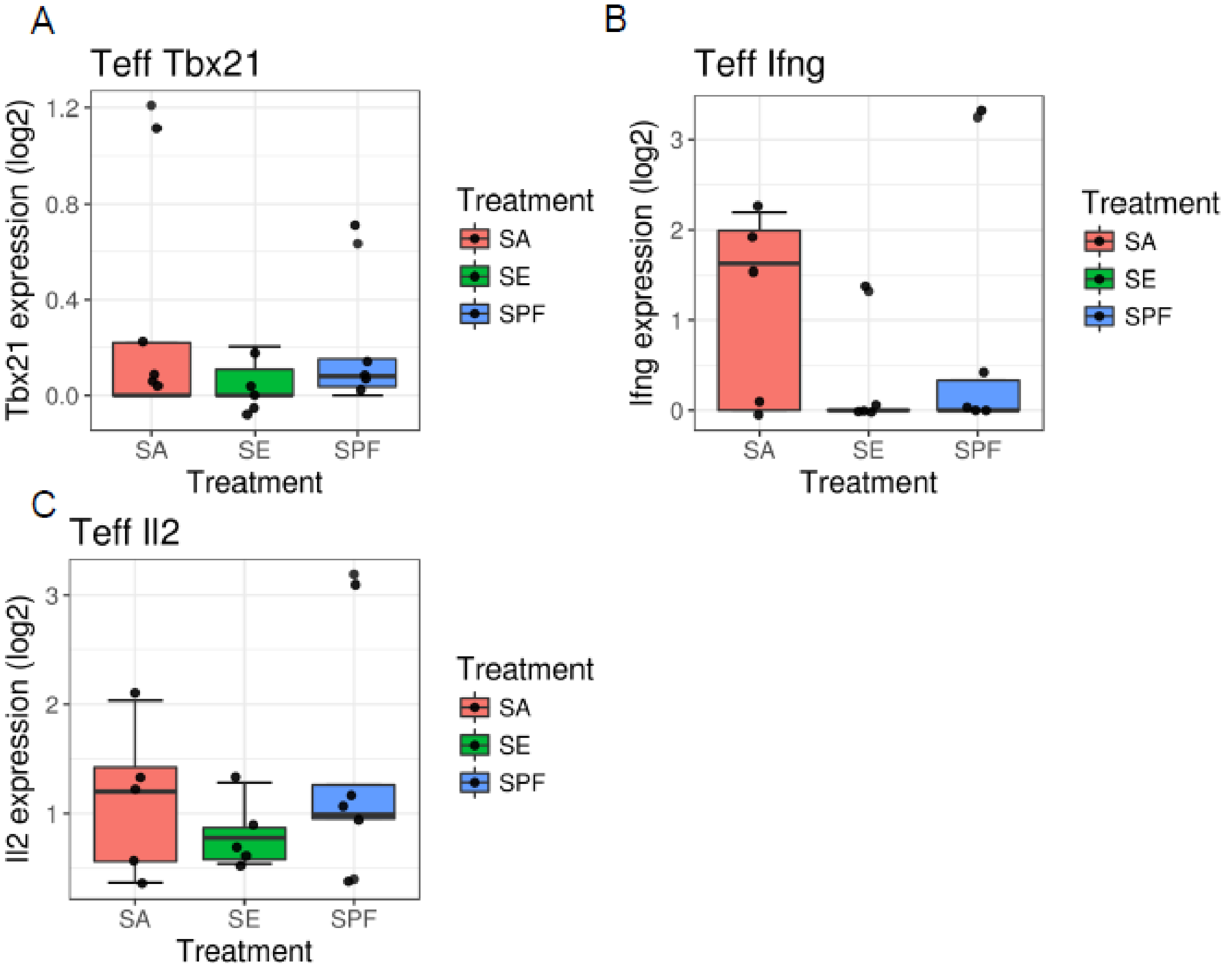

Supplementary Figure 5

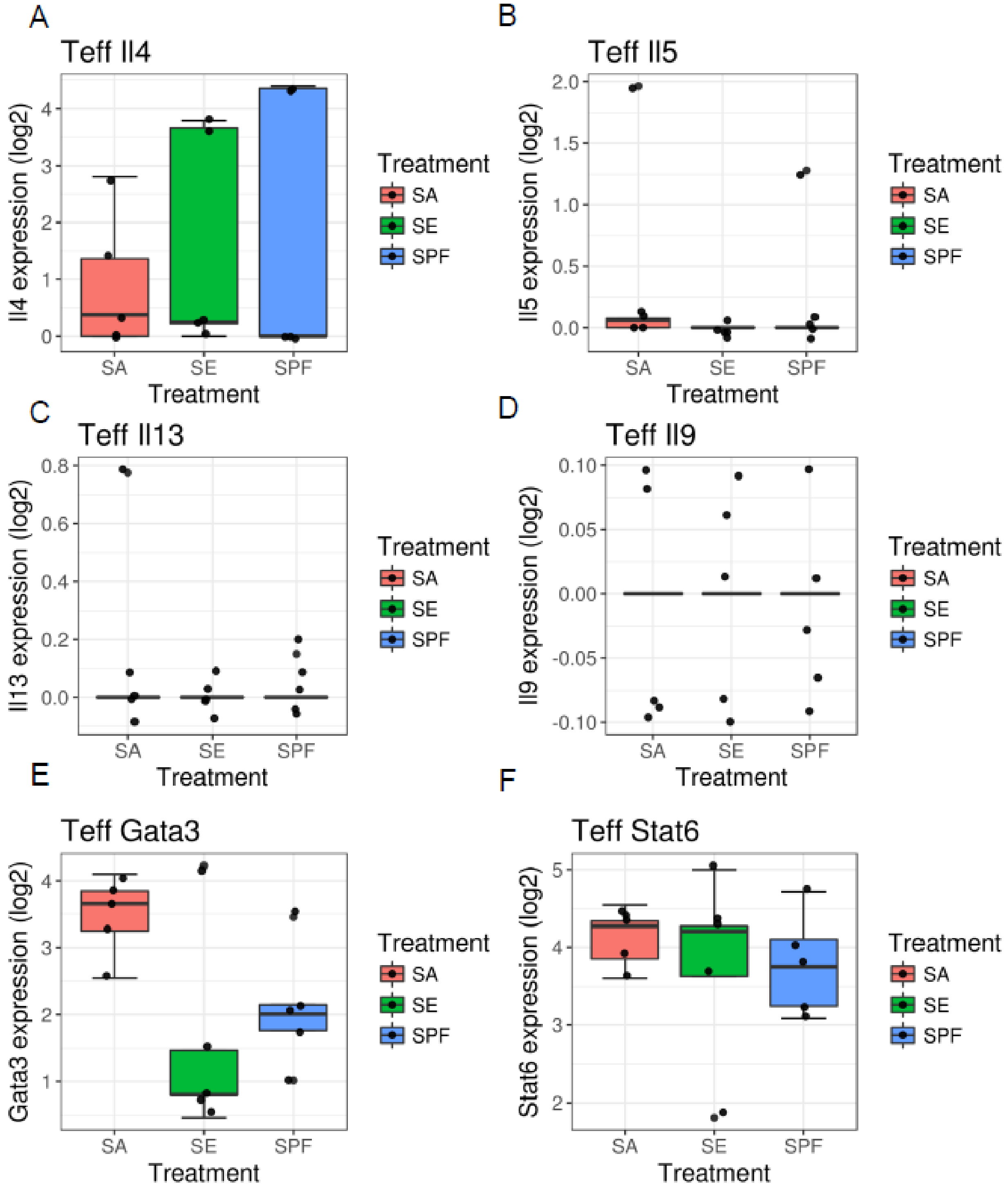

Supplement: Supplementary file 1 — Figure S1. Box plot shows the average weighted UniFrac distances among samples within each disease state at (a) arm, (b)trunk, (c)leg, (d)axilla, (e)gluteal fold and (f)scalp (*: p-value < 0.05, **: p-value < 0.01***: p-value < 0.001****: p-value < 0.0001). Figure S2. Box plot shows the average weighted UniFrac distances among samples within each disease state in (a) dry and (b)moist skin group (*: p-value < 0.05, **: p-value < 0.01***: p-value < 0.001****: p-value < 0.0001). Figure S3. Relative abundance of P. acnes and S. aureus in each disease state at different body sites. Relative abundance of P. acnes in healthy (red), psoriasis lesional (green), psoriasis non-lesional (blue) skin at (a) different skin sites and (b) different skin types (*: p-val < 0.05, **: p-val < 0.01, ****: p-val < 0.0001). (c) Box plot showing S. aureus abundance in S. aureus high samples. S. aureus high samples were defined as samples with higher S. aureus abundance than the highest S. aureus abundance among the healthy samples (baseline = 0.0068). (d) Bar graph depicts the prevalence of S. aureus high samples at each skin site in psoriasis lesional (blue bars) and psoriasis non-lesional (orange bars) skin. (e) Bacterial species associated with S. aureus high samples (red bars) and S. aureus low samples (green bars). Figure S4. Expression of Th1 components in effector T cells in response to Staphylococcus aureus colonization. The expression of Th1 components (a) T-bet (b) IFNγ and (c)IL-2 are comparable in all experimental groups. Figure S5. Expression of Th2 components in effector T cells in response to Staphylococcus aureus colonization. The expression of Th2 cytokines (a) IL-4 (b) IL-5, (c) IL-13 (d) IL-9 are comparable in all experimental groups. The expression of Th2 promoting transcription factor (e) GATA3 is induced by early colonization of S. aureus (adj. p-value = 1.49e-16); whereas, another Th2 transcription factor (f) STAT6 is not significantly induced. (PDF 405 kb) [file 40168_2018_533_MOESM1_ESM.pdf]
