# Supplementary material for: Alteration of the cutaneous microbiome in psoriasis and potential role in Th17 polarization
Source: Microbiome. 2018 Sep 5;6:154. doi: 10.1186/s40168-018-0533-1 (PMC6125946; doi:10.1186/s40168-018-0533-1)
Supplement: Supplementary file 2 — R notebook for applying linear mixed effects model to test the significance of difference in microbial diversity among psoriatic lesional, psoriatic unaffected, and healthy skin. (HTML 772 kb) [file 40168_2018_533_MOESM2_ESM.html]

Running linear mixed effect model


Code 

- Show All Code
- Hide All Code
- Download Rmd

# Running linear mixed effect model

This is an R Markdown Notebook. When you execute code within the notebook, the results appear beneath the code.

Try executing this chunk by clicking the *Run* button within the chunk or by placing your cursor inside it and pressing *Ctrl+Shift+Enter*.


```
library(lmerTest)
map <- read.table("/mnt/ultrastarQuatre/Microbiome/Skin/skin_pool/skin1234567/No_Ear_Face/Alpha_Diversity_11766_Depth/final_map.txt", sep = '\t', header=TRUE)
choa1_file <- read.table("/mnt/ultrastarQuatre/Microbiome/Skin/skin_pool/skin1234567/No_Ear_Face/Alpha_Diversity_11766_Depth/chao1.txt", sep = '\t', header =TRUE)
simpson_file <- read.table("/mnt/ultrastarQuatre/Microbiome/Skin/skin_pool/skin1234567/No_Ear_Face/Alpha_Diversity_11766_Depth/simpson.txt", sep = '\t', header =TRUE)
```


merge the two tables


```
mdata <- merge(map,choa1_file,by="SampleID")
mdata <- merge(mdata,simpson_file, by="SampleID")
mdata <- mdata[,c(1,5,9,10,13,14)]
mdata <- merge(mdata, beta_file, by="SampleID")
```


LME with Healthy as reference


```
mdata$Treatment <- factor(mdata$Treatment,levels=c("Healthy","PSO_L","PSO_N"))
### Chao1 stats (reference=Healthy)
chao1.model.healthy = lmer(Chao1 ~ Treatment + (1|Patient) , data=mdata)
summary(chao1.model.healthy)
```


```
Linear mixed model fit by REML 
t-tests use  Satterthwaite approximations to degrees of freedom ['lmerMod']
Formula: Chao1 ~ Treatment + (1 | Patient)
   Data: mdata

REML criterion at convergence: 6441.2

Scaled residuals: 
    Min      1Q  Median      3Q     Max 
-2.3497 -0.5633 -0.0451  0.4410  4.3364 

Random effects:
 Groups   Name        Variance Std.Dev.
 Patient  (Intercept) 102521   320.2   
 Residual             272936   522.4   
Number of obs: 417, groups:  Patient, 54

Fixed effects:
               Estimate Std. Error     df t value Pr(>|t|)    
(Intercept)      888.58      76.24  63.58  11.654   <2e-16 ***
TreatmentPSO_L   168.07     109.55  72.24   1.534    0.129    
TreatmentPSO_N   124.73     105.90  63.64   1.178    0.243    
---
Signif. codes:  0 ‘***’ 0.001 ‘**’ 0.01 ‘*’ 0.05 ‘.’ 0.1 ‘ ’ 1

Correlation of Fixed Effects:
            (Intr) TPSO_L
TrtmntPSO_L -0.696       
TrtmntPSO_N -0.720  0.817
```


```
### Simpson stats (reference= Healthy)
simpson.model.healthy = lmer(Simpson ~Treatment +(1|Patient), data=mdata)
summary(simpson.model.healthy)
```


```
Linear mixed model fit by REML 
t-tests use  Satterthwaite approximations to degrees of freedom ['lmerMod']
Formula: Simpson ~ Treatment + (1 | Patient)
   Data: mdata

REML criterion at convergence: -265.2

Scaled residuals: 
    Min      1Q  Median      3Q     Max 
-4.1327 -0.3715  0.2769  0.5856  1.8460 

Random effects:
 Groups   Name        Variance Std.Dev.
 Patient  (Intercept) 0.00963  0.09813 
 Residual             0.02513  0.15852 
Number of obs: 417, groups:  Patient, 54

Fixed effects:
               Estimate Std. Error       df t value Pr(>|t|)    
(Intercept)     0.77381    0.02329 59.82000  33.222   <2e-16 ***
TreatmentPSO_L  0.05284    0.03345 67.95000   1.579    0.119    
TreatmentPSO_N  0.03621    0.03235 59.87000   1.119    0.268    
---
Signif. codes:  0 ‘***’ 0.001 ‘**’ 0.01 ‘*’ 0.05 ‘.’ 0.1 ‘ ’ 1

Correlation of Fixed Effects:
            (Intr) TPSO_L
TrtmntPSO_L -0.696       
TrtmntPSO_N -0.720  0.819
```


```
### Beta-diversity (PC1) stats (reference= Healthy)
beta.pc1.healthy = lmer(PC1 ~ Treatment + (1|Patient), data = mdata)
summary(beta.pc1.healthy)
```


```
Linear mixed model fit by REML 
t-tests use  Satterthwaite approximations to degrees of freedom ['lmerMod']
Formula: PC1 ~ Treatment + (1 | Patient)
   Data: mdata

REML criterion at convergence: -348.4

Scaled residuals: 
     Min       1Q   Median       3Q      Max 
-2.54785 -0.62132 -0.03505  0.67839  2.97771 

Random effects:
 Groups   Name        Variance Std.Dev.
 Patient  (Intercept) 0.008044 0.08969 
 Residual             0.020505 0.14319 
Number of obs: 417, groups:  Patient, 54

Fixed effects:
               Estimate Std. Error       df t value Pr(>|t|)
(Intercept)     0.03222    0.02121 61.44000   1.519    0.134
TreatmentPSO_L -0.04946    0.03045 69.63000  -1.624    0.109
TreatmentPSO_N -0.04547    0.02946 61.49000  -1.543    0.128

Correlation of Fixed Effects:
            (Intr) TPSO_L
TrtmntPSO_L -0.697       
TrtmntPSO_N -0.720  0.822
```


RME using PSO\_N as reference


```
mdata$Treatment <- factor(mdata$Treatment,levels=c("PSO_N","PSO_L","Healthy"))
### Chao1 stats (refence = PSO_N)
chao1.model.PSO_N = lmer(Chao1 ~ Treatment + (1|Patient) , data=mdata)
summary(chao1.model.PSO_N)
```


```
Linear mixed model fit by REML 
t-tests use  Satterthwaite approximations to degrees of freedom ['lmerMod']
Formula: Chao1 ~ Treatment + (1 | Patient)
   Data: mdata

REML criterion at convergence: 6441.2

Scaled residuals: 
    Min      1Q  Median      3Q     Max 
-2.3497 -0.5633 -0.0451  0.4410  4.3364 

Random effects:
 Groups   Name        Variance Std.Dev.
 Patient  (Intercept) 102521   320.2   
 Residual             272936   522.4   
Number of obs: 417, groups:  Patient, 54

Fixed effects:
                 Estimate Std. Error      df t value Pr(>|t|)    
(Intercept)       1013.31      73.49   63.70  13.788   <2e-16 ***
TreatmentPSO_L      43.34      65.30  372.20   0.664    0.507    
TreatmentHealthy  -124.73     105.90   63.60  -1.178    0.243    
---
Signif. codes:  0 ‘***’ 0.001 ‘**’ 0.01 ‘*’ 0.05 ‘.’ 0.1 ‘ ’ 1

Correlation of Fixed Effects:
            (Intr) TPSO_L
TrtmntPSO_L -0.362       
TrtmntHlthy -0.694  0.251
```


```
### Simpson stats (reference= PSO_N)
simpson.model.PSO_N = lmer(Simpson ~Treatment +(1|Patient), data=mdata)
summary(simpson.model.PSO_N)
```


```
Linear mixed model fit by REML 
t-tests use  Satterthwaite approximations to degrees of freedom ['lmerMod']
Formula: Simpson ~ Treatment + (1 | Patient)
   Data: mdata

REML criterion at convergence: -265.2

Scaled residuals: 
    Min      1Q  Median      3Q     Max 
-4.1327 -0.3715  0.2769  0.5856  1.8460 

Random effects:
 Groups   Name        Variance Std.Dev.
 Patient  (Intercept) 0.00963  0.09813 
 Residual             0.02513  0.15852 
Number of obs: 417, groups:  Patient, 54

Fixed effects:
                  Estimate Std. Error        df t value Pr(>|t|)    
(Intercept)        0.81002    0.02245  59.90000  36.078   <2e-16 ***
TreatmentPSO_L     0.01663    0.01981 367.50000   0.839    0.402    
TreatmentHealthy  -0.03621    0.03235  59.90000  -1.119    0.268    
---
Signif. codes:  0 ‘***’ 0.001 ‘**’ 0.01 ‘*’ 0.05 ‘.’ 0.1 ‘ ’ 1

Correlation of Fixed Effects:
            (Intr) TPSO_L
TrtmntPSO_L -0.360       
TrtmntHlthy -0.694  0.250
```


```
### Beta-diversity (PC1) stats (reference= PSO_N)
beta.pc1.PSO_N = lmer(PC1 ~ Treatment + (1|Patient), data = mdata)
summary(beta.pc1.PSO_N)
```


```
Linear mixed model fit by REML 
t-tests use  Satterthwaite approximations to degrees of freedom ['lmerMod']
Formula: PC1 ~ Treatment + (1 | Patient)
   Data: mdata

REML criterion at convergence: -348.4

Scaled residuals: 
     Min       1Q   Median       3Q      Max 
-2.54785 -0.62132 -0.03505  0.67839  2.97771 

Random effects:
 Groups   Name        Variance Std.Dev.
 Patient  (Intercept) 0.008044 0.08969 
 Residual             0.020505 0.14319 
Number of obs: 417, groups:  Patient, 54

Fixed effects:
                   Estimate Std. Error         df t value Pr(>|t|)
(Intercept)       -0.013249   0.020445  61.600000  -0.648    0.519
TreatmentPSO_L    -0.003989   0.017900 368.800000  -0.223    0.824
TreatmentHealthy   0.045469   0.029460  61.500000   1.543    0.128

Correlation of Fixed Effects:
            (Intr) TPSO_L
TrtmntPSO_L -0.357       
TrtmntHlthy -0.694  0.248
```


LS0tCnRpdGxlOiAiUnVubmluZyBsaW5lYXIgbWl4ZWQgZWZmZWN0IG1vZGVsIgpvdXRwdXQ6CiAgaHRtbF9kb2N1bWVudDoKICAgIGRmX3ByaW50OiBwYWdlZAogIGh0bWxfbm90ZWJvb2s6IGRlZmF1bHQKICBwZGZfZG9jdW1lbnQ6IGRlZmF1bHQKLS0tCgpUaGlzIGlzIGFuIFtSIE1hcmtkb3duXShodHRwOi8vcm1hcmtkb3duLnJzdHVkaW8uY29tKSBOb3RlYm9vay4gV2hlbiB5b3UgZXhlY3V0ZSBjb2RlIHdpdGhpbiB0aGUgbm90ZWJvb2ssIHRoZSByZXN1bHRzIGFwcGVhciBiZW5lYXRoIHRoZSBjb2RlLiAKClRyeSBleGVjdXRpbmcgdGhpcyBjaHVuayBieSBjbGlja2luZyB0aGUgKlJ1biogYnV0dG9uIHdpdGhpbiB0aGUgY2h1bmsgb3IgYnkgcGxhY2luZyB5b3VyIGN1cnNvciBpbnNpZGUgaXQgYW5kIHByZXNzaW5nICpDdHJsK1NoaWZ0K0VudGVyKi4gCgpgYGB7cn0KbGlicmFyeShsbWVyVGVzdCkKbWFwIDwtIHJlYWQudGFibGUoIi9tbnQvdWx0cmFzdGFyUXVhdHJlL01pY3JvYmlvbWUvU2tpbi9za2luX3Bvb2wvc2tpbjEyMzQ1NjcvTm9fRWFyX0ZhY2UvQWxwaGFfRGl2ZXJzaXR5XzExNzY2X0RlcHRoL2ZpbmFsX21hcC50eHQiLCBzZXAgPSAnXHQnLCBoZWFkZXI9VFJVRSkKY2hvYTFfZmlsZSA8LSByZWFkLnRhYmxlKCIvbW50L3VsdHJhc3RhclF1YXRyZS9NaWNyb2Jpb21lL1NraW4vc2tpbl9wb29sL3NraW4xMjM0NTY3L05vX0Vhcl9GYWNlL0FscGhhX0RpdmVyc2l0eV8xMTc2Nl9EZXB0aC9jaGFvMS50eHQiLCBzZXAgPSAnXHQnLCBoZWFkZXIgPVRSVUUpCnNpbXBzb25fZmlsZSA8LSByZWFkLnRhYmxlKCIvbW50L3VsdHJhc3RhclF1YXRyZS9NaWNyb2Jpb21lL1NraW4vc2tpbl9wb29sL3NraW4xMjM0NTY3L05vX0Vhcl9GYWNlL0FscGhhX0RpdmVyc2l0eV8xMTc2Nl9EZXB0aC9zaW1wc29uLnR4dCIsIHNlcCA9ICdcdCcsIGhlYWRlciA9VFJVRSkKCmJldGFfZmlsZSA8LSByZWFkLnRhYmxlKCIvbW50L3VsdHJhc3RhclF1YXRyZS9NaWNyb2Jpb21lL1NraW4vc2tpbl9wb29sL3NraW4xMjM0NTY3L05vX0Vhcl9GYWNlL0JldGFfRGl2ZXJzaXR5L3dlaWdodGVkX3VuaWZyYWNfUENzLnR4dCIsIHNlcCA9ICdcdCcsIGhlYWRlciA9VFJVRSkKYGBgCm1lcmdlIHRoZSB0d28gdGFibGVzCgpgYGB7cn0KCm1kYXRhIDwtIG1lcmdlKG1hcCxjaG9hMV9maWxlLGJ5PSJTYW1wbGVJRCIpCm1kYXRhIDwtIG1lcmdlKG1kYXRhLHNpbXBzb25fZmlsZSwgYnk9IlNhbXBsZUlEIikKbWRhdGEgPC0gbWRhdGFbLGMoMSw1LDksMTAsMTMsMTQpXQptZGF0YSA8LSBtZXJnZShtZGF0YSwgYmV0YV9maWxlLCBieT0iU2FtcGxlSUQiKQpgYGAKTE1FIHdpdGggSGVhbHRoeSBhcyByZWZlcmVuY2UKYGBge3J9Cm1kYXRhJFRyZWF0bWVudCA8LSBmYWN0b3IobWRhdGEkVHJlYXRtZW50LGxldmVscz1jKCJIZWFsdGh5IiwiUFNPX0wiLCJQU09fTiIpKQojIyMgQ2hhbzEgc3RhdHMgKHJlZmVyZW5jZT1IZWFsdGh5KQpjaGFvMS5tb2RlbC5oZWFsdGh5ID0gbG1lcihDaGFvMSB+IFRyZWF0bWVudCArICgxfFBhdGllbnQpICwgZGF0YT1tZGF0YSkKc3VtbWFyeShjaGFvMS5tb2RlbC5oZWFsdGh5KQoKIyMjIFNpbXBzb24gc3RhdHMgKHJlZmVyZW5jZT0gSGVhbHRoeSkKc2ltcHNvbi5tb2RlbC5oZWFsdGh5ID0gbG1lcihTaW1wc29uIH5UcmVhdG1lbnQgKygxfFBhdGllbnQpLCBkYXRhPW1kYXRhKQpzdW1tYXJ5KHNpbXBzb24ubW9kZWwuaGVhbHRoeSkKCiMjIyBCZXRhLWRpdmVyc2l0eSAoUEMxKSBzdGF0cyAocmVmZXJlbmNlPSBIZWFsdGh5KQpiZXRhLnBjMS5oZWFsdGh5ID0gbG1lcihQQzEgfiBUcmVhdG1lbnQgKyAoMXxQYXRpZW50KSwgZGF0YSA9IG1kYXRhKQpzdW1tYXJ5KGJldGEucGMxLmhlYWx0aHkpCgoKYGBgClJNRSB1c2luZyBQU09fTiBhcyByZWZlcmVuY2UKYGBge3J9Cm1kYXRhJFRyZWF0bWVudCA8LSBmYWN0b3IobWRhdGEkVHJlYXRtZW50LGxldmVscz1jKCJQU09fTiIsIlBTT19MIiwiSGVhbHRoeSIpKQojIyMgQ2hhbzEgc3RhdHMgKHJlZmVuY2UgPSBQU09fTikKY2hhbzEubW9kZWwuUFNPX04gPSBsbWVyKENoYW8xIH4gVHJlYXRtZW50ICsgKDF8UGF0aWVudCkgLCBkYXRhPW1kYXRhKQpzdW1tYXJ5KGNoYW8xLm1vZGVsLlBTT19OKQojIyMgU2ltcHNvbiBzdGF0cyAocmVmZXJlbmNlPSBQU09fTikKc2ltcHNvbi5tb2RlbC5QU09fTiA9IGxtZXIoU2ltcHNvbiB+VHJlYXRtZW50ICsoMXxQYXRpZW50KSwgZGF0YT1tZGF0YSkKc3VtbWFyeShzaW1wc29uLm1vZGVsLlBTT19OKQoKIyMjIEJldGEtZGl2ZXJzaXR5IChQQzEpIHN0YXRzIChyZWZlcmVuY2U9IFBTT19OKQpiZXRhLnBjMS5QU09fTiA9IGxtZXIoUEMxIH4gVHJlYXRtZW50ICsgKDF8UGF0aWVudCksIGRhdGEgPSBtZGF0YSkKc3VtbWFyeShiZXRhLnBjMS5QU09fTikKYGBg
