# Supplementary material for: Alteration of the cutaneous microbiome in psoriasis and potential role in Th17 polarization
Source: Microbiome. 2018 Sep 5;6:154. doi: 10.1186/s40168-018-0533-1 (PMC6125946; doi:10.1186/s40168-018-0533-1)
Supplement: Supplementary file 4 — R notebook for microbe-microbe correlation analyses at genus and species level. (HTML 1518 kb) [file 40168_2018_533_MOESM4_ESM.html]

Skin Microbiome correlation analysis


Code 

- Show All Code
- Hide All Code
- Download Rmd

# Skin Microbiome correlation analysis

This is an R Markdown Notebook. When you execute code within the notebook, the results appear beneath the code.


```
library(Hmisc)
```


```
Loading required package: lattice
Loading required package: survival
Loading required package: Formula

Attaching package: ‘Hmisc’

The following objects are masked from ‘package:base’:

    format.pval, round.POSIXt, trunc.POSIXt, units
```


```
library(ggcorrplot)
```


Microbe-microbe correaltion at the genus level (L6)


```
#load taxonomy summary table at genus level (L6) generated by Qiime
skin1234567_allgenera<-read.table("/mnt/ultrastarQuatre/Microbiome/Skin/skin_pool/skin1234567/skin1234567_allgenera_stripped.csv",sep = ',',header = T,row.names = 1)
#transpose table and change all columns except the status column to numeric
skin1234567_allgenera <- data.frame(t(skin1234567_allgenera))
skin1234567_allgenera<-data.frame(lapply(skin1234567_allgenera, function(x) as.numeric(as.character(x))))
skin1234567_allgenera["Sum" ,]<-colSums(skin1234567_allgenera)
#sorted dataframe by sum of abundance and take top 25 most abundant genera 
skin1234567_allgenera_sorted <- skin1234567_allgenera[,order(-skin1234567_allgenera[418,])]
top25genera<-skin1234567_allgenera_sorted[,c(1:25)]
top25genera<-top25genera[-418,]
#calculate spearman correlation and p value
correlation_genera<-round(cor(top25genera,method = "spearman"),1)
p.mat.genera <- cor_pmat(top25genera)
ggcorrplot(correlation_genera, hc.order = TRUE, p.mat = p.mat.genera, insig = "blank",
   outline.col = "white",
   ggtheme = ggplot2::theme_gray,
   colors = c("#6D9EC1", "white", "#E46726"))
```


```
#ggsave("Spearman_Corr_plot_top25genera.tiff",height = 10, width = 10, units = 'in', dpi=300)
```


Microbe-microbe correaltion at the species level (L7)


```
#load Taxonomy summary species table (L7) generated by Qiime (Sum was calculated in excel)
skin1234567_allspp<-read.table("/mnt/ultrastarQuatre/Microbiome/Skin/skin_pool/skin1234567/skin1234567_all_spp_stripped.csv",sep = ',',header = T,row.names = 1)
#transpose table and change all columns except the status column to numeric
skin1234567_allspp <- data.frame(t(skin1234567_allspp))
skin1234567_allspp[,c(2:217)]<-lapply(skin1234567_allspp[,c(2:217)], function(x) as.numeric(as.character(x)))
#sorted dataframe by sum of abundance and take top 30 most abundant spp. 
skin1234567_allspp_sorted <- skin1234567_allspp[,order(-skin1234567_allspp[418,])]
```


```
‘-’ not meaningful for factors
```


```
top30spp<-skin1234567_allspp_sorted[,c(217,1:30)]
colnames(top30spp)<- gsub("\\."," ", colnames(top30spp))
```


correlation of microbes for all samples


```
top30spp_all<-top30spp[-418,-1]
correlation_all<-round(cor(top30spp_all,method = "spearman"),1)
p.mat.all <- cor_pmat(top30spp_all)
ggcorrplot(correlation_all, hc.order = TRUE, p.mat = p.mat.all, insig = "blank",
   outline.col = "white",
   ggtheme = ggplot2::theme_gray,
   colors = c("#6D9EC1", "white", "#E46726"))
```


```
#ggsave("Spearman_Corr_plot_top30spp_All.tiff",height = 10, width = 10, units = 'in', dpi=300)
```


Calculating spearman’s correlation among bacteria spp in psoriasis lesional (PSOL) samples


```
#subsetting PSOL
PSOL_rows <- top30spp[,1] == "PSOL"
top30spp_PSOL <- top30spp[PSOL_rows,]
top30spp_PSOL <- top30spp_PSOL[,-1]
#Calculate spearman correlations and p values 
correlation_PSOL<-round(cor(top30spp_PSOL,method = "spearman"),1)
p.mat.PSOL <- cor_pmat(top30spp_PSOL)
ggcorrplot(correlation_PSOL, hc.order = TRUE, p.mat = p.mat.PSOL, insig = "blank",
   outline.col = "white",
   ggtheme = ggplot2::theme_gray,
   colors = c("#6D9EC1", "white", "#E46726"))
ggsave("Spearman_Corr_plot_top30spp_PSOL-only.tiff",height = 10, width = 10, units = 'in', dpi=300)
```


Calculating spearman’s correlation among bacteria spp in psoriasis normal (PSON) samples


```
#subsetting PSON
PSON_rows <- top30spp[,1] == "PSON"
top30spp_PSON <- top30spp[PSON_rows,]
top30spp_PSON <- top30spp_PSON[,-1]
#Calculate spearman correlations and p values 
correlation_PSON<-round(cor(top30spp_PSON,method = "spearman"),1)
p.mat.PSON <- cor_pmat(top30spp_PSON)
ggcorrplot(correlation_PSON, hc.order = TRUE, p.mat = p.mat.PSON, insig = "blank",
   outline.col = "white",
   ggtheme = ggplot2::theme_gray,
   colors = c("#6D9EC1", "white", "#E46726"))
```


```
#ggsave("Spearman_Corr_plot_top30spp_PSON-only.tiff",height = 10, width = 10, units = 'in', dpi=300)
```


Calculating spearman’s correlation among bacteria spp in Healthy (H) samples


```
#subsetting healthy samples
H_rows <- top30spp[,1] == "Healthy"
top30spp_H <- top30spp[H_rows,]
top30spp_H <- top30spp_H[,-1]
#Calculate spearman correlations and p values 
correlation_H<-round(cor(top30spp_H,method = "spearman"),1)
p.mat.H <- cor_pmat(top30spp_H)
ggcorrplot(correlation_H, hc.order = TRUE, p.mat = p.mat.H, insig = "blank",
   outline.col = "white",
   ggtheme = ggplot2::theme_gray,
   colors = c("#6D9EC1", "white", "#E46726"))
```


```
#ggsave("Spearman_Corr_plot_top30spp_PSOL-only.tiff",height = 10, width = 10, units = 'in', dpi=300)
```


LS0tCnRpdGxlOiAiU2tpbiBNaWNyb2Jpb21lIGNvcnJlbGF0aW9uIGFuYWx5c2lzIgpvdXRwdXQ6CiAgcGRmX2RvY3VtZW50OiBkZWZhdWx0CiAgaHRtbF9ub3RlYm9vazogZGVmYXVsdAotLS0KClRoaXMgaXMgYW4gW1IgTWFya2Rvd25dKGh0dHA6Ly9ybWFya2Rvd24ucnN0dWRpby5jb20pIE5vdGVib29rLiBXaGVuIHlvdSBleGVjdXRlIGNvZGUgd2l0aGluIHRoZSBub3RlYm9vaywgdGhlIHJlc3VsdHMgYXBwZWFyIGJlbmVhdGggdGhlIGNvZGUuIAoKCmBgYHtyfQpsaWJyYXJ5KEhtaXNjKQpsaWJyYXJ5KGdnY29ycnBsb3QpCgpgYGAKTWljcm9iZS1taWNyb2JlIGNvcnJlYWx0aW9uIGF0IHRoZSBnZW51cyBsZXZlbCAoTDYpCmBgYHtyfQojbG9hZCB0YXhvbm9teSBzdW1tYXJ5IHRhYmxlIGF0IGdlbnVzIGxldmVsIChMNikgZ2VuZXJhdGVkIGJ5IFFpaW1lCnNraW4xMjM0NTY3X2FsbGdlbmVyYTwtcmVhZC50YWJsZSgiL21udC91bHRyYXN0YXJRdWF0cmUvTWljcm9iaW9tZS9Ta2luL3NraW5fcG9vbC9za2luMTIzNDU2Ny9za2luMTIzNDU2N19hbGxnZW5lcmFfc3RyaXBwZWQuY3N2IixzZXAgPSAnLCcsaGVhZGVyID0gVCxyb3cubmFtZXMgPSAxKQoKI3RyYW5zcG9zZSB0YWJsZSBhbmQgY2hhbmdlIGFsbCBjb2x1bW5zIGV4Y2VwdCB0aGUgc3RhdHVzIGNvbHVtbiB0byBudW1lcmljCgpza2luMTIzNDU2N19hbGxnZW5lcmEgPC0gZGF0YS5mcmFtZSh0KHNraW4xMjM0NTY3X2FsbGdlbmVyYSkpCnNraW4xMjM0NTY3X2FsbGdlbmVyYTwtZGF0YS5mcmFtZShsYXBwbHkoc2tpbjEyMzQ1NjdfYWxsZ2VuZXJhLCBmdW5jdGlvbih4KSBhcy5udW1lcmljKGFzLmNoYXJhY3Rlcih4KSkpKQoKc2tpbjEyMzQ1NjdfYWxsZ2VuZXJhWyJTdW0iICxdPC1jb2xTdW1zKHNraW4xMjM0NTY3X2FsbGdlbmVyYSkKCiNzb3J0ZWQgZGF0YWZyYW1lIGJ5IHN1bSBvZiBhYnVuZGFuY2UgYW5kIHRha2UgdG9wIDI1IG1vc3QgYWJ1bmRhbnQgZ2VuZXJhIApza2luMTIzNDU2N19hbGxnZW5lcmFfc29ydGVkIDwtIHNraW4xMjM0NTY3X2FsbGdlbmVyYVssb3JkZXIoLXNraW4xMjM0NTY3X2FsbGdlbmVyYVs0MTgsXSldCnRvcDI1Z2VuZXJhPC1za2luMTIzNDU2N19hbGxnZW5lcmFfc29ydGVkWyxjKDE6MjUpXQp0b3AyNWdlbmVyYTwtdG9wMjVnZW5lcmFbLTQxOCxdCiNjYWxjdWxhdGUgc3BlYXJtYW4gY29ycmVsYXRpb24gYW5kIHAgdmFsdWUKY29ycmVsYXRpb25fZ2VuZXJhPC1yb3VuZChjb3IodG9wMjVnZW5lcmEsbWV0aG9kID0gInNwZWFybWFuIiksMSkKcC5tYXQuZ2VuZXJhIDwtIGNvcl9wbWF0KHRvcDI1Z2VuZXJhKQpnZ2NvcnJwbG90KGNvcnJlbGF0aW9uX2dlbmVyYSwgaGMub3JkZXIgPSBUUlVFLCBwLm1hdCA9IHAubWF0LmdlbmVyYSwgaW5zaWcgPSAiYmxhbmsiLAogICBvdXRsaW5lLmNvbCA9ICJ3aGl0ZSIsCiAgIGdndGhlbWUgPSBnZ3Bsb3QyOjp0aGVtZV9ncmF5LAogICBjb2xvcnMgPSBjKCIjNkQ5RUMxIiwgIndoaXRlIiwgIiNFNDY3MjYiKSkKI2dnc2F2ZSgiU3BlYXJtYW5fQ29ycl9wbG90X3RvcDI1Z2VuZXJhLnRpZmYiLGhlaWdodCA9IDEwLCB3aWR0aCA9IDEwLCB1bml0cyA9ICdpbicsIGRwaT0zMDApCgpgYGAKCgpNaWNyb2JlLW1pY3JvYmUgY29ycmVhbHRpb24gYXQgdGhlIHNwZWNpZXMgbGV2ZWwgKEw3KQpgYGB7cn0KI2xvYWQgVGF4b25vbXkgc3VtbWFyeSBzcGVjaWVzIHRhYmxlIChMNykgZ2VuZXJhdGVkIGJ5IFFpaW1lIChTdW0gd2FzIGNhbGN1bGF0ZWQgaW4gZXhjZWwpCnNraW4xMjM0NTY3X2FsbHNwcDwtcmVhZC50YWJsZSgiL21udC91bHRyYXN0YXJRdWF0cmUvTWljcm9iaW9tZS9Ta2luL3NraW5fcG9vbC9za2luMTIzNDU2Ny9za2luMTIzNDU2N19hbGxfc3BwX3N0cmlwcGVkLmNzdiIsc2VwID0gJywnLGhlYWRlciA9IFQscm93Lm5hbWVzID0gMSkKCiN0cmFuc3Bvc2UgdGFibGUgYW5kIGNoYW5nZSBhbGwgY29sdW1ucyBleGNlcHQgdGhlIHN0YXR1cyBjb2x1bW4gdG8gbnVtZXJpYwpza2luMTIzNDU2N19hbGxzcHAgPC0gZGF0YS5mcmFtZSh0KHNraW4xMjM0NTY3X2FsbHNwcCkpCnNraW4xMjM0NTY3X2FsbHNwcFssYygyOjIxNyldPC1sYXBwbHkoc2tpbjEyMzQ1NjdfYWxsc3BwWyxjKDI6MjE3KV0sIGZ1bmN0aW9uKHgpIGFzLm51bWVyaWMoYXMuY2hhcmFjdGVyKHgpKSkKCiNzb3J0ZWQgZGF0YWZyYW1lIGJ5IHN1bSBvZiBhYnVuZGFuY2UgYW5kIHRha2UgdG9wIDMwIG1vc3QgYWJ1bmRhbnQgc3BwLiAKc2tpbjEyMzQ1NjdfYWxsc3BwX3NvcnRlZCA8LSBza2luMTIzNDU2N19hbGxzcHBbLG9yZGVyKC1za2luMTIzNDU2N19hbGxzcHBbNDE4LF0pXQp0b3AzMHNwcDwtc2tpbjEyMzQ1NjdfYWxsc3BwX3NvcnRlZFssYygyMTcsMTozMCldCmNvbG5hbWVzKHRvcDMwc3BwKTwtIGdzdWIoIlxcLiIsIiAiLCBjb2xuYW1lcyh0b3AzMHNwcCkpCmBgYApjb3JyZWxhdGlvbiBvZiBtaWNyb2JlcyBmb3IgYWxsIHNhbXBsZXMKYGBge3J9Cgp0b3AzMHNwcF9hbGw8LXRvcDMwc3BwWy00MTgsLTFdCmNvcnJlbGF0aW9uX2FsbDwtcm91bmQoY29yKHRvcDMwc3BwX2FsbCxtZXRob2QgPSAic3BlYXJtYW4iKSwxKQpwLm1hdC5hbGwgPC0gY29yX3BtYXQodG9wMzBzcHBfYWxsKQpnZ2NvcnJwbG90KGNvcnJlbGF0aW9uX2FsbCwgaGMub3JkZXIgPSBUUlVFLCBwLm1hdCA9IHAubWF0LmFsbCwgaW5zaWcgPSAiYmxhbmsiLAogICBvdXRsaW5lLmNvbCA9ICJ3aGl0ZSIsCiAgIGdndGhlbWUgPSBnZ3Bsb3QyOjp0aGVtZV9ncmF5LAogICBjb2xvcnMgPSBjKCIjNkQ5RUMxIiwgIndoaXRlIiwgIiNFNDY3MjYiKSkKI2dnc2F2ZSgiU3BlYXJtYW5fQ29ycl9wbG90X3RvcDMwc3BwX0FsbC50aWZmIixoZWlnaHQgPSAxMCwgd2lkdGggPSAxMCwgdW5pdHMgPSAnaW4nLCBkcGk9MzAwKQoKCmBgYApDYWxjdWxhdGluZyBzcGVhcm1hbidzIGNvcnJlbGF0aW9uIGFtb25nIGJhY3RlcmlhIHNwcCBpbiBwc29yaWFzaXMgbGVzaW9uYWwgKFBTT0wpIHNhbXBsZXMKYGBge3J9CiNzdWJzZXR0aW5nIFBTT0wKUFNPTF9yb3dzIDwtIHRvcDMwc3BwWywxXSA9PSAiUFNPTCIKdG9wMzBzcHBfUFNPTCA8LSB0b3AzMHNwcFtQU09MX3Jvd3MsXQp0b3AzMHNwcF9QU09MIDwtIHRvcDMwc3BwX1BTT0xbLC0xXQoKI0NhbGN1bGF0ZSBzcGVhcm1hbiBjb3JyZWxhdGlvbnMgYW5kIHAgdmFsdWVzIApjb3JyZWxhdGlvbl9QU09MPC1yb3VuZChjb3IodG9wMzBzcHBfUFNPTCxtZXRob2QgPSAic3BlYXJtYW4iKSwxKQpwLm1hdC5QU09MIDwtIGNvcl9wbWF0KHRvcDMwc3BwX1BTT0wpCmdnY29ycnBsb3QoY29ycmVsYXRpb25fUFNPTCwgaGMub3JkZXIgPSBUUlVFLCBwLm1hdCA9IHAubWF0LlBTT0wsIGluc2lnID0gImJsYW5rIiwKICAgb3V0bGluZS5jb2wgPSAid2hpdGUiLAogICBnZ3RoZW1lID0gZ2dwbG90Mjo6dGhlbWVfZ3JheSwKICAgY29sb3JzID0gYygiIzZEOUVDMSIsICJ3aGl0ZSIsICIjRTQ2NzI2IikpCmdnc2F2ZSgiU3BlYXJtYW5fQ29ycl9wbG90X3RvcDMwc3BwX1BTT0wtb25seS50aWZmIixoZWlnaHQgPSAxMCwgd2lkdGggPSAxMCwgdW5pdHMgPSAnaW4nLCBkcGk9MzAwKQoKYGBgCgoKCkNhbGN1bGF0aW5nIHNwZWFybWFuJ3MgY29ycmVsYXRpb24gYW1vbmcgYmFjdGVyaWEgc3BwIGluIHBzb3JpYXNpcyBub3JtYWwgKFBTT04pIHNhbXBsZXMKYGBge3J9CiNzdWJzZXR0aW5nIFBTT04KUFNPTl9yb3dzIDwtIHRvcDMwc3BwWywxXSA9PSAiUFNPTiIKdG9wMzBzcHBfUFNPTiA8LSB0b3AzMHNwcFtQU09OX3Jvd3MsXQp0b3AzMHNwcF9QU09OIDwtIHRvcDMwc3BwX1BTT05bLC0xXQojQ2FsY3VsYXRlIHNwZWFybWFuIGNvcnJlbGF0aW9ucyBhbmQgcCB2YWx1ZXMgCmNvcnJlbGF0aW9uX1BTT048LXJvdW5kKGNvcih0b3AzMHNwcF9QU09OLG1ldGhvZCA9ICJzcGVhcm1hbiIpLDEpCnAubWF0LlBTT04gPC0gY29yX3BtYXQodG9wMzBzcHBfUFNPTikKZ2djb3JycGxvdChjb3JyZWxhdGlvbl9QU09OLCBoYy5vcmRlciA9IFRSVUUsIHAubWF0ID0gcC5tYXQuUFNPTiwgaW5zaWcgPSAiYmxhbmsiLAogICBvdXRsaW5lLmNvbCA9ICJ3aGl0ZSIsCiAgIGdndGhlbWUgPSBnZ3Bsb3QyOjp0aGVtZV9ncmF5LAogICBjb2xvcnMgPSBjKCIjNkQ5RUMxIiwgIndoaXRlIiwgIiNFNDY3MjYiKSkKI2dnc2F2ZSgiU3BlYXJtYW5fQ29ycl9wbG90X3RvcDMwc3BwX1BTT04tb25seS50aWZmIixoZWlnaHQgPSAxMCwgd2lkdGggPSAxMCwgdW5pdHMgPSAnaW4nLCBkcGk9MzAwKQoKYGBgCgpDYWxjdWxhdGluZyBzcGVhcm1hbidzIGNvcnJlbGF0aW9uIGFtb25nIGJhY3RlcmlhIHNwcCBpbiBIZWFsdGh5IChIKSBzYW1wbGVzCmBgYHtyfQojc3Vic2V0dGluZyBoZWFsdGh5IHNhbXBsZXMKSF9yb3dzIDwtIHRvcDMwc3BwWywxXSA9PSAiSGVhbHRoeSIKdG9wMzBzcHBfSCA8LSB0b3AzMHNwcFtIX3Jvd3MsXQp0b3AzMHNwcF9IIDwtIHRvcDMwc3BwX0hbLC0xXQojQ2FsY3VsYXRlIHNwZWFybWFuIGNvcnJlbGF0aW9ucyBhbmQgcCB2YWx1ZXMgCmNvcnJlbGF0aW9uX0g8LXJvdW5kKGNvcih0b3AzMHNwcF9ILG1ldGhvZCA9ICJzcGVhcm1hbiIpLDEpCnAubWF0LkggPC0gY29yX3BtYXQodG9wMzBzcHBfSCkKZ2djb3JycGxvdChjb3JyZWxhdGlvbl9ILCBoYy5vcmRlciA9IFRSVUUsIHAubWF0ID0gcC5tYXQuSCwgaW5zaWcgPSAiYmxhbmsiLAogICBvdXRsaW5lLmNvbCA9ICJ3aGl0ZSIsCiAgIGdndGhlbWUgPSBnZ3Bsb3QyOjp0aGVtZV9ncmF5LAogICBjb2xvcnMgPSBjKCIjNkQ5RUMxIiwgIndoaXRlIiwgIiNFNDY3MjYiKSkKI2dnc2F2ZSgiU3BlYXJtYW5fQ29ycl9wbG90X3RvcDMwc3BwX1BTT0wtb25seS50aWZmIixoZWlnaHQgPSAxMCwgd2lkdGggPSAxMCwgdW5pdHMgPSAnaW4nLCBkcGk9MzAwKQpgYGAKCgoK
