# Supplementary material for: Alteration of the cutaneous microbiome in psoriasis and potential role in Th17 polarization
Source: Microbiome. 2018 Sep 5;6:154. doi: 10.1186/s40168-018-0533-1 (PMC6125946; doi:10.1186/s40168-018-0533-1)
Supplement: Supplementary file 7 — Mann-Kendall Test to detect trend in alpha diversity. (HTML 785 kb) [file 40168_2018_533_MOESM7_ESM.html]

Mann-Kendall Test to detect trend in alpha diveristy


# Mann-Kendall Test to detect trend in alpha diveristy

```
install.packages("Kendall")
```

```
## Installing package into '/home/ellachang/R/x86_64-pc-linux-gnu-library/3.3'
## (as 'lib' is unspecified)
```

```
require(Kendall)
```

```
## Loading required package: Kendall
```

```
load("/mnt/ultrastarQuatre/Microbiome/Skin/skin_pool/skin1234567/final_otu_by_Sites/Alpha_div_by_sites/skin1234567_sites-specific_alpha_diversity_stats_LME.rdata")
```

Mann-Kendall test to test trend in alpha diversity with all skin sites combined

```
### adding numeric ID to the disease status: Healthy(1), PSO_N(2), PSO_L(3)
map$TreatmentID<-gsub("Healthy",1,map$Treatment)
map$TreatmentID<-gsub("PSO_N",2,map$TreatmentID)
map$TreatmentID<-gsub("PSO_L",3,map$TreatmentID)

meta<-map[,c("SampleID","Treatment","Sites","Skin_Type","TreatmentID")]

all_skin_diveristy<-merge(meta,chao1)
all_skin_diveristy<-merge(all_skin_diveristy,otu)
all_skin_diveristy<-merge(all_skin_diveristy,shannon)
all_skin_diveristy<-merge(all_skin_diveristy,simpson)
all_skin_diveristy$TreatmentID<-all_skin_diveristy$Treatment
all_skin_diveristy$TreatmentID<-gsub("Healthy",1,all_skin_diveristy$TreatmentID)
all_skin_diveristy$TreatmentID<-gsub("PSO_N",2,all_skin_diveristy$TreatmentID)
all_skin_diveristy$TreatmentID<-gsub("PSO_L",3,all_skin_diveristy$TreatmentID)

print("Mann-Kendall Test to detech trend in chao1")
```

```
## [1] "Mann-Kendall Test to detech trend in chao1"
```

```
Kendall(all_skin_diveristy$chao1,all_skin_diveristy$TreatmentID)
```

```
## tau = -0.0509, 2-sided pvalue =0.17839
```

```
print("Mann-Kendall Test to detech trend in observed otus")
```

```
## [1] "Mann-Kendall Test to detech trend in observed otus"
```

```
Kendall(all_skin_diveristy$otu,all_skin_diveristy$TreatmentID)
```

```
## tau = 0.0544, 2-sided pvalue =0.15079
```

```
print("Mann-Kendall Test to detech trend in shannon")
```

```
## [1] "Mann-Kendall Test to detech trend in shannon"
```

```
Kendall(all_skin_diveristy$shannon,all_skin_diveristy$TreatmentID)
```

```
## tau = 0.106, 2-sided pvalue =0.0052354
```

```
print("Mann-Kendall Test to detech trend in simpson")
```

```
## [1] "Mann-Kendall Test to detech trend in simpson"
```

```
Kendall(all_skin_diveristy$simpson,all_skin_diveristy$TreatmentID)
```

```
## tau = 0.125, 2-sided pvalue =0.00096667
```

Mann-Kendall test to test trend in alpha diversity with Arm only

```
Arm_chao1<-data.frame(t(Arm_chao1))
Arm_chao1$SampleID<-row.names(Arm_chao1)
Arm_OTU<-data.frame(t(Arm_OTU))
Arm_OTU$SampleID<-row.names(Arm_OTU)
Arm_shannon<-data.frame(t(Arm_shannon))
Arm_shannon$SampleID<-row.names(Arm_shannon)
Arm_simpson<-data.frame(t(Arm_simpson))
Arm_simpson$SampleID<-row.names(Arm_simpson)


Arm_diveristy<-merge(meta,Arm_chao1)
Arm_diveristy<-merge(Arm_diveristy,Arm_OTU)
Arm_diveristy<-merge(Arm_diveristy,Arm_shannon)
Arm_diveristy<-merge(Arm_diveristy,Arm_simpson)

print("Mann-Kendall Test to detech trend in arm chao1")
```

```
## [1] "Mann-Kendall Test to detech trend in arm chao1"
```

```
Kendall(Arm_diveristy$Arm.chao1,Arm_diveristy$TreatmentID)
```

```
## tau = 0.119, 2-sided pvalue =0.19024
```

```
print("Mann-Kendall Test to detech trend in arm observed otus")
```

```
## [1] "Mann-Kendall Test to detech trend in arm observed otus"
```

```
Kendall(Arm_diveristy$Arm.otu,Arm_diveristy$TreatmentID)
```

```
## tau = 0.143, 2-sided pvalue =0.11462
```

```
print("Mann-Kendall Test to detech trend in arm shannon")
```

```
## [1] "Mann-Kendall Test to detech trend in arm shannon"
```

```
Kendall(Arm_diveristy$Arm.shannon,Arm_diveristy$TreatmentID)
```

```
## tau = 0.242, 2-sided pvalue =0.007511
```

```
print("Mann-Kendall Test to detech trend in arm simpson")
```

```
## [1] "Mann-Kendall Test to detech trend in arm simpson"
```

```
Kendall(Arm_diveristy$Arm.simpson,Arm_diveristy$TreatmentID)
```

```
## tau = 0.28, 2-sided pvalue =0.0019234
```

Mann-Kendall test to test trend in alpha diversity with Ax only

```
Axilla_chao1<-data.frame(t(Axilla_chao1))
Axilla_chao1$SampleID<-row.names(Axilla_chao1)
Axilla_OTU<-data.frame(t(Axilla_OTU))
Axilla_OTU$SampleID<-row.names(Axilla_OTU)
Axilla_shannon<-data.frame(t(Axilla_shannon))
Axilla_shannon$SampleID<-row.names(Axilla_shannon)
Axilla_simpson<-data.frame(t(Axilla_simpson))
Axilla_simpson$SampleID<-row.names(Axilla_simpson)


Axilla_diveristy<-merge(meta,Axilla_chao1)
Axilla_diveristy<-merge(Axilla_diveristy,Axilla_OTU)
Axilla_diveristy<-merge(Axilla_diveristy,Axilla_shannon)
Axilla_diveristy<-merge(Axilla_diveristy,Axilla_simpson)

print("Mann-Kendall Test to detech trend in Axilla chao1")
```

```
## [1] "Mann-Kendall Test to detech trend in Axilla chao1"
```

```
Kendall(Axilla_diveristy$Axilla.chao1,Axilla_diveristy$TreatmentID)
```

```
## tau = 0.0158, 2-sided pvalue =0.89391
```

```
print("Mann-Kendall Test to detech trend in Axilla observed otus")
```

```
## [1] "Mann-Kendall Test to detech trend in Axilla observed otus"
```

```
Kendall(Axilla_diveristy$Axilla.otu,Axilla_diveristy$TreatmentID)
```

```
## tau = 0.0267, 2-sided pvalue =0.81718
```

```
print("Mann-Kendall Test to detech trend in Axilla shannon")
```

```
## [1] "Mann-Kendall Test to detech trend in Axilla shannon"
```

```
Kendall(Axilla_diveristy$Axilla.shannon,Axilla_diveristy$TreatmentID)
```

```
## tau = 0.149, 2-sided pvalue =0.18526
```

```
print("Mann-Kendall Test to detech trend in Axilla simpson")
```

```
## [1] "Mann-Kendall Test to detech trend in Axilla simpson"
```

```
Kendall(Axilla_diveristy$Axilla.simpson,Axilla_diveristy$TreatmentID)
```

```
## tau = 0.139, 2-sided pvalue =0.21652
```

Mann-Kendall test to test trend in alpha diversity with gluteal fold only

```
Butt_Crease_chao1<-data.frame(t(Butt_Crease_chao1))
Butt_Crease_chao1$SampleID<-row.names(Butt_Crease_chao1)
Butt_Crease_OTU<-data.frame(t(Butt_Crease_OTU))
Butt_Crease_OTU$SampleID<-row.names(Butt_Crease_OTU)
Butt_Crease_shannon<-data.frame(t(Butt_Crease_shannon))
Butt_Crease_shannon$SampleID<-row.names(Butt_Crease_shannon)
Butt_Crease_simpson<-data.frame(t(Butt_Crease_simpson))
Butt_Crease_simpson$SampleID<-row.names(Butt_Crease_simpson)


Butt_Crease_diveristy<-merge(meta,Butt_Crease_chao1)
Butt_Crease_diveristy<-merge(Butt_Crease_diveristy,Butt_Crease_OTU)
Butt_Crease_diveristy<-merge(Butt_Crease_diveristy,Butt_Crease_shannon)
Butt_Crease_diveristy<-merge(Butt_Crease_diveristy,Butt_Crease_simpson)

print("Mann-Kendall Test to detech trend in gluteal fold chao1")
```

```
## [1] "Mann-Kendall Test to detech trend in gluteal fold chao1"
```

```
Kendall(Butt_Crease_diveristy$Butt_Crease.chao1,Butt_Crease_diveristy$TreatmentID)
```

```
## tau = 0.0262, 2-sided pvalue =0.7892
```

```
print("Mann-Kendall Test to detech trend in gluteal fold  observed otus")
```

```
## [1] "Mann-Kendall Test to detech trend in gluteal fold  observed otus"
```

```
Kendall(Butt_Crease_diveristy$Butt_Crease.otu,Butt_Crease_diveristy$TreatmentID)
```

```
## tau = 0.019, 2-sided pvalue =0.8479
```

```
print("Mann-Kendall Test to detech trend in gluteal fold  shannon")
```

```
## [1] "Mann-Kendall Test to detech trend in gluteal fold  shannon"
```

```
Kendall(Butt_Crease_diveristy$Butt_Crease.shannon,Butt_Crease_diveristy$TreatmentID)
```

```
## tau = 0.0117, 2-sided pvalue =0.90746
```

```
print("Mann-Kendall Test to detech trend in gluteal fold m simpson")
```

```
## [1] "Mann-Kendall Test to detech trend in gluteal fold m simpson"
```

```
Kendall(Butt_Crease_diveristy$Butt_Crease.simpson,Butt_Crease_diveristy$TreatmentID)
```

```
## tau = 0.0808, 2-sided pvalue =0.40264
```

Mann-Kendall test to test trend in alpha diversity with Leg only

```
Leg_chao1<-data.frame(t(Leg_chao1))
Leg_chao1$SampleID<-row.names(Leg_chao1)
Leg_OTU<-data.frame(t(Leg_OTU))
Leg_OTU$SampleID<-row.names(Leg_OTU)
Leg_shannon<-data.frame(t(Leg_shannon))
Leg_shannon$SampleID<-row.names(Leg_shannon)
Leg_simpson<-data.frame(t(Leg_simpson))
Leg_simpson$SampleID<-row.names(Leg_simpson)


Leg_diveristy<-merge(meta,Leg_chao1)
Leg_diveristy<-merge(Leg_diveristy,Leg_OTU)
Leg_diveristy<-merge(Leg_diveristy,Leg_shannon)
Leg_diveristy<-merge(Leg_diveristy,Leg_simpson)

print("Mann-Kendall Test to detech trend in Leg chao1")
```

```
## [1] "Mann-Kendall Test to detech trend in Leg chao1"
```

```
Kendall(Leg_diveristy$Leg.chao1,Leg_diveristy$TreatmentID)
```

```
## tau = 0.0729, 2-sided pvalue =0.40272
```

```
print("Mann-Kendall Test to detech trend in Leg observed otus")
```

```
## [1] "Mann-Kendall Test to detech trend in Leg observed otus"
```

```
Kendall(Leg_diveristy$Leg.otu,Leg_diveristy$TreatmentID)
```

```
## tau = 0.0782, 2-sided pvalue =0.36946
```

```
print("Mann-Kendall Test to detech trend in Leg shannon")
```

```
## [1] "Mann-Kendall Test to detech trend in Leg shannon"
```

```
Kendall(Leg_diveristy$Leg.shannon,Leg_diveristy$TreatmentID)
```

```
## tau = 0.0466, 2-sided pvalue =0.59384
```

```
print("Mann-Kendall Test to detech trend in Leg simpson")
```

```
## [1] "Mann-Kendall Test to detech trend in Leg simpson"
```

```
Kendall(Leg_diveristy$Leg.simpson,Leg_diveristy$TreatmentID)
```

```
## tau = 0.0541, 2-sided pvalue =0.53526
```

Mann-Kendall test to test trend in alpha diversity with Scalp only

```
Scalp_chao1<-data.frame(t(Scalp_chao1))
Scalp_chao1$SampleID<-row.names(Scalp_chao1)
Scalp_OTU<-data.frame(t(Scalp_OTU))
Scalp_OTU$SampleID<-row.names(Scalp_OTU)
Scalp_shannon<-data.frame(t(Scalp_shannon))
Scalp_shannon$SampleID<-row.names(Scalp_shannon)
Scalp_simpson<-data.frame(t(Scalp_simpson))
Scalp_simpson$SampleID<-row.names(Scalp_simpson)


Scalp_diveristy<-merge(meta,Scalp_chao1)
Scalp_diveristy<-merge(Scalp_diveristy,Scalp_OTU)
Scalp_diveristy<-merge(Scalp_diveristy,Scalp_shannon)
Scalp_diveristy<-merge(Scalp_diveristy,Scalp_simpson)

print("Mann-Kendall Test to detech trend in Scalp chao1")
```

```
## [1] "Mann-Kendall Test to detech trend in Scalp chao1"
```

```
Kendall(Scalp_diveristy$Scalp.chao1,Scalp_diveristy$TreatmentID)
```

```
## tau = 0.171, 2-sided pvalue =0.062372
```

```
print("Mann-Kendall Test to detech trend in Scalp observed otus")
```

```
## [1] "Mann-Kendall Test to detech trend in Scalp observed otus"
```

```
Kendall(Scalp_diveristy$Scalp.otu,Scalp_diveristy$TreatmentID)
```

```
## tau = 0.164, 2-sided pvalue =0.074637
```

```
print("Mann-Kendall Test to detech trend in Scalp shannon")
```

```
## [1] "Mann-Kendall Test to detech trend in Scalp shannon"
```

```
Kendall(Scalp_diveristy$Scalp.shannon,Scalp_diveristy$TreatmentID)
```

```
## tau = 0.104, 2-sided pvalue =0.25663
```

```
print("Mann-Kendall Test to detech trend in Scalp simpson")
```

```
## [1] "Mann-Kendall Test to detech trend in Scalp simpson"
```

```
Kendall(Scalp_diveristy$Scalp.simpson,Scalp_diveristy$TreatmentID)
```

```
## tau = 0.0671, 2-sided pvalue =0.46585
```

Mann-Kendall test to test trend in alpha diversity with Trunk only

```
Trunk_chao1<-data.frame(t(Trunk_chao1))
Trunk_chao1$SampleID<-row.names(Trunk_chao1)
Trunk_OTU<-data.frame(t(Trunk_OTU))
Trunk_OTU$SampleID<-row.names(Trunk_OTU)
Trunk_shannon<-data.frame(t(Trunk_shannon))
Trunk_shannon$SampleID<-row.names(Trunk_shannon)
Trunk_simpson<-data.frame(t(Trunk_simpson))
Trunk_simpson$SampleID<-row.names(Trunk_simpson)


Trunk_diveristy<-merge(meta,Trunk_chao1)
Trunk_diveristy<-merge(Trunk_diveristy,Trunk_OTU)
Trunk_diveristy<-merge(Trunk_diveristy,Trunk_shannon)
Trunk_diveristy<-merge(Trunk_diveristy,Trunk_simpson)

print("Mann-Kendall Test to detech trend in Trunk chao1")
```

```
## [1] "Mann-Kendall Test to detech trend in Trunk chao1"
```

```
Kendall(Trunk_diveristy$Trunk.chao1,Trunk_diveristy$TreatmentID)
```

```
## tau = 0.101, 2-sided pvalue =0.28262
```

```
print("Mann-Kendall Test to detech trend in Trunk observed otus")
```

```
## [1] "Mann-Kendall Test to detech trend in Trunk observed otus"
```

```
Kendall(Trunk_diveristy$Trunk.otu,Trunk_diveristy$TreatmentID)
```

```
## tau = 0.129, 2-sided pvalue =0.17147
```

```
print("Mann-Kendall Test to detech trend in Trunk shannon")
```

```
## [1] "Mann-Kendall Test to detech trend in Trunk shannon"
```

```
Kendall(Trunk_diveristy$Trunk.shannon,Trunk_diveristy$TreatmentID)
```

```
## tau = 0.157, 2-sided pvalue =0.094651
```

```
print("Mann-Kendall Test to detech trend in Trunk simpson")
```

```
## [1] "Mann-Kendall Test to detech trend in Trunk simpson"
```

```
Kendall(Trunk_diveristy$Trunk.simpson,Trunk_diveristy$TreatmentID)
```

```
## tau = 0.168, 2-sided pvalue =0.073335
```

Mann-Kendall test to test trend in alpha diversity with dry only

```
dry_chao1<-data.frame(t(dry_chao1))
dry_chao1$SampleID<-row.names(dry_chao1)
dry_OTU<-data.frame(t(dry_OTU))
dry_OTU$SampleID<-row.names(dry_OTU)
dry_shannon<-data.frame(t(dry_shannon))
dry_shannon$SampleID<-row.names(dry_shannon)
dry_simpson<-data.frame(t(dry_simpson))
dry_simpson$SampleID<-row.names(dry_simpson)


dry_diveristy<-merge(meta,dry_chao1)
dry_diveristy<-merge(dry_diveristy,dry_OTU)
dry_diveristy<-merge(dry_diveristy,dry_shannon)
dry_diveristy<-merge(dry_diveristy,dry_simpson)

print("Mann-Kendall Test to detech trend in dry chao1")
```

```
## [1] "Mann-Kendall Test to detech trend in dry chao1"
```

```
Kendall(dry_diveristy$dry.chao1,dry_diveristy$TreatmentID)
```

```
## tau = 0.1, 2-sided pvalue =0.051078
```

```
print("Mann-Kendall Test to detech trend in dry observed otus")
```

```
## [1] "Mann-Kendall Test to detech trend in dry observed otus"
```

```
Kendall(dry_diveristy$dry.otu,dry_diveristy$TreatmentID)
```

```
## tau = 0.118, 2-sided pvalue =0.021886
```

```
print("Mann-Kendall Test to detech trend in dry shannon")
```

```
## [1] "Mann-Kendall Test to detech trend in dry shannon"
```

```
Kendall(dry_diveristy$dry.shannon,dry_diveristy$TreatmentID)
```

```
## tau = 0.148, 2-sided pvalue =0.0041585
```

```
print("Mann-Kendall Test to detech trend in dry simpson")
```

```
## [1] "Mann-Kendall Test to detech trend in dry simpson"
```

```
Kendall(dry_diveristy$dry.simpson,dry_diveristy$TreatmentID)
```

```
## tau = 0.169, 2-sided pvalue =0.0010333
```

Mann-Kendall test to test trend in alpha diversity with moist only

```
moist_chao1<-data.frame(t(moist_chao1))
moist_chao1$SampleID<-row.names(moist_chao1)
moist_OTU<-data.frame(t(moist_OTU))
moist_OTU$SampleID<-row.names(moist_OTU)
moist_shannon<-data.frame(t(moist_shannon))
moist_shannon$SampleID<-row.names(moist_shannon)
moist_simpson<-data.frame(t(moist_simpson))
moist_simpson$SampleID<-row.names(moist_simpson)


moist_diveristy<-merge(meta,moist_chao1)
moist_diveristy<-merge(moist_diveristy,moist_OTU)
moist_diveristy<-merge(moist_diveristy,moist_shannon)
moist_diveristy<-merge(moist_diveristy,moist_simpson)

print("Mann-Kendall Test to detech trend in moist chao1")
```

```
## [1] "Mann-Kendall Test to detech trend in moist chao1"
```

```
Kendall(moist_diveristy$moist.chao1,moist_diveristy$TreatmentID)
```

```
## tau = 0.00995, 2-sided pvalue =0.8921
```

```
print("Mann-Kendall Test to detech trend in moist observed otus")
```

```
## [1] "Mann-Kendall Test to detech trend in moist observed otus"
```

```
Kendall(moist_diveristy$moist.otu,moist_diveristy$TreatmentID)
```

```
## tau = 0.0128, 2-sided pvalue =0.86042
```

```
print("Mann-Kendall Test to detech trend in moist shannon")
```

```
## [1] "Mann-Kendall Test to detech trend in moist shannon"
```

```
Kendall(moist_diveristy$moist.shannon,moist_diveristy$TreatmentID)
```

```
## tau = 0.0479, 2-sided pvalue =0.50723
```

```
print("Mann-Kendall Test to detech trend in moist simpson")
```

```
## [1] "Mann-Kendall Test to detech trend in moist simpson"
```

```
Kendall(moist_diveristy$moist.simpson,moist_diveristy$TreatmentID)
```

```
## tau = 0.0834, 2-sided pvalue =0.24788
```

```
save.image("/mnt/ultrastarQuatre/Microbiome/Skin/skin_pool/skin1234567/final_otu_by_Sites/Alpha_div_by_sites/skin_Mann-Kendall_trend_test.rdata")
```
